# Supplementary material for: Phase-matching-free sensing with undetected light using a nonlinear thin-film metasurface
Source: Nat Commun. 2026 Aug 22;17:10117. doi: 10.1038/s41467-026-76512-5 (PMC13598124; doi:10.1038/s41467-026-76512-5)
Supplement: Supplementary file 1 [file 41467_2026_76512_MOESM1_ESM.pdf]

Supplementary Information: Phase-matching-free sensing with undetected light using a nonlinear thin-film metasurface

Toby Severs Millard[1,2], Nathan R. Gemmell[1], Ross C. Schofield[1], Mohsen Rahmani[3], Alex S. Clark[1,4], Chris C. Phillips[1], and Rupert F. Oulton[1]

[1]Blackett Laboratory, Department of Physics, Imperial College London, London SW7 2AZ, UK
[2]Quantum Materials Group, Quantum Technologies Department, National Physical Laboratory, Teddington TW11 0LW, UK
[3]Advanced Optics & Photonics Laboratory, Department of Engineering, School of Science & Technology, Nottingham Trent University, Nottingham NG11 8NS, United Kingdom
[4]Quantum Engineering Technology Labs, H. H. Wills Physics Laboratory and School of Electrical, Electronic and Mechanical Engineering, University of Bristol, BS8 1FD, UK.

Supplementary Note I. Experimental optical parameters

Transmission and reflection measurements were taken with the same tunable pulsed laser used for the main experiment at 835 nm and 1500 nm for the pump and seed (idler), respectively. For measurements at 580 nm, the seed was tuned to 1160 nm and passed through a frequency doubler. The FWM (signal) emission in each direction, $\sigma_\pm$, was extracted from the intensities at the camera shown in Supplementary Fig. 4(a) and (b) (Fig. 1(g) and (h) of the main text), using constants from Supplementary Table I. The visibility in Supplementary Table I was calculated from all extracted parameters using Eq. (7) of the main text (Eq. (S20) below) and represents an average across the detector.

Parameter	Label	Value	Uncertainty (±)
Metasurface pump transmission	T_p	0.784	0.002
Metasurface seed transmission	T_s	0.47	0.01
Metasurface FWM transmission	T_f	0.80	0.03
Metasurface FWM reflection	R_f	0.118	0.002
Normalised FWM emission in + direction	σ_+	0.530	0.002
Normalised FWM emission in - direction	σ_-	0.469	0.001
FWM emission ratio (σ_-/σ_+)	η	0.885	0.004
Average visibility	V	0.54	0.01

Supplementary Table I. Experimentally measured optical parameters of the metasurface used to calculate visibility.

Supplementary Note II.   Analytical visibility derivation

At the first pass through the metasurface, the amplitude and phase of the pump and seed fields are described in the frequency domain, respectively, by

$$E_{p1} = E_p(\omega)e^{i\phi_p(\omega)} \tag{S1}$$

and

$$E_{s1} = E_s(\omega)e^{i\phi_s(\omega)}. \tag{S2}$$

Here, $E_{p,s}(\omega)$ and $\phi_{p,s}(\omega)$ are the frequency dependent amplitude and phase of the pump and seed. After travelling the paths shown in Supplementary Fig. 1, the fields at the second pass become

$$E_{p2} = t_p E_{p1} e^{2ik_p d} \tag{S3}$$

and

$$E_{s2} = t_s t e^{i\gamma} E_{s1} e^{2ik_s d'}. \tag{S4}$$

Where $t_{p,s}$ are the pump and seed transmission amplitudes through the metasurface, and it is assumed the dichroic mirror is perfectly reflective for the pump and FWM, and is perfectly transmissive for the seed (idler). The seed (idler) beam passes through an object or material at O with transmission t, inducing a phase shift of γ.

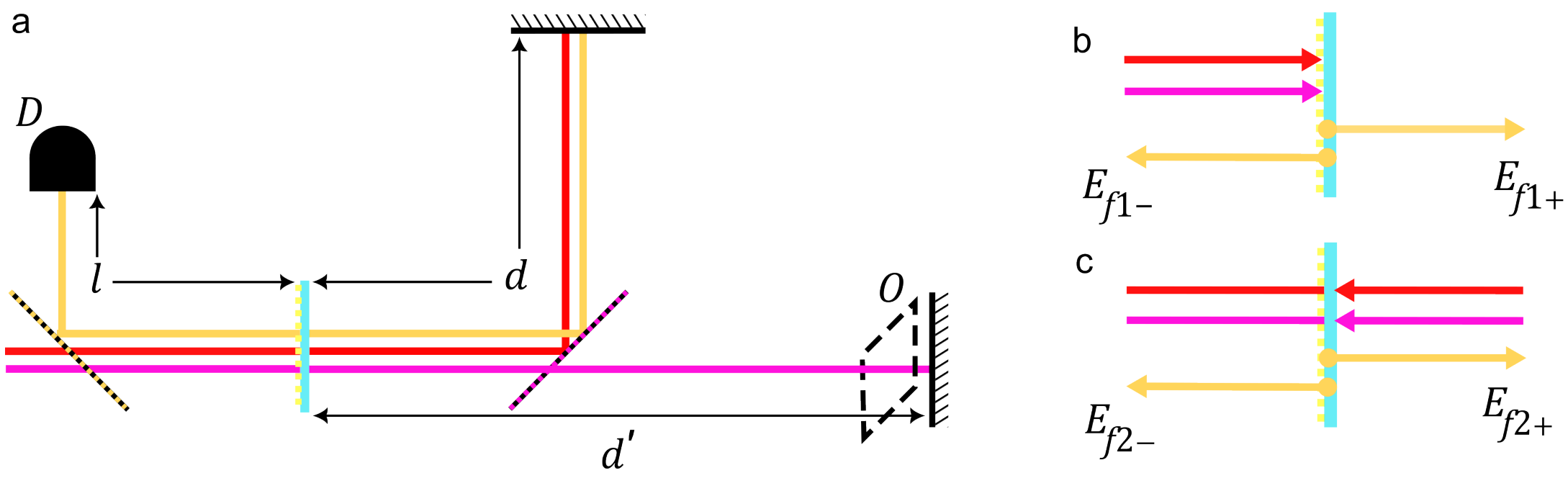

Supplementary Figure 1. A simplified schematic of the interferometer system shown in Fig. 1(a) of the main text to support the derivation below. (a) Beam paths of the pump (red), seed (idler, magenta) and FWM (signal, yellow) with the path lengths labelled. Due to the relaxation of out-of-plane phase matching requirements, FWM signal is emitted in both the + and − directions at the (b) first and (c) second pass. This, combined with pulsed illumination, creates temporal windows where interference can occur.

Due to the subwavelength thickness, the phase matching requirements are relaxed and FWM (signal) is emitted out of the metasurface plane in both directions. However, due to the different refractive indices of the sub- and superstrate, the strength of emission is not equal in each direction relative to the metasurface (+ and -). We define FWM (signal) by the input beams

$$E_{f1\pm,f2\pm} = \alpha_\pm E^2_{p1,p2} E^*_{s1,p2} \tag{S5}$$

where $\alpha_\pm$ indicates the strength of the nonlinear process, which is related to the nonlinear $\chi^{(3)}_{\mu xxy}$ tensor elements for the process. We are working under the assumption of undepleted beams, such that after

FWM generation the pump and seed amplitudes are unchanged.

FWM fields generated at each pass and each direction are then evaluated at the detector using Eqs. (S3-S5) and path distances within the interferometer.

$$E_{f1-} = \alpha_- E_{p1}^2 E_{s1}^* e^{ik_f l} \tag{S6}$$

$$E_{f1+} = \alpha_+ t_f E_{p1}^2 E_{s1}^* e^{ik_f (2d+l)} \tag{S7}$$

$$E_{f2-} = \alpha_- E_{p2}^2 E_{s2}^* e^{ik_f l} = \alpha_- t e^{i\gamma} t_p^2 t_s E_{p1}^2 E_{s1}^* e^{ik_f l - 2ik_s d' + 4ik_p d} \tag{S8}$$

$$E_{f2+} = \alpha_+ E_{p2}^2 E_{s2}^* e^{ik_f (2d+l)} = \alpha_+ t_f t e^{i\gamma} t_p^2 t_s E_{p1}^2 E_{s1}^* e^{ik_f (2d+l) - 2ik_s d' + 4ik_p d} \tag{S9}$$

Here, t_f is the transmission amplitude of the metasurface at the FWM wavelength. To simplify the above equations, we introduce the average nonlinear amplitude in each direction at either pass, $\sigma_\pm$,

$$\alpha_\pm E_p^2 E_s^* e^{ik_f l} e^{i(2\phi_p(\omega) - \phi_s(\omega))} = \tilde{\sigma}_\pm(\omega) e^{i\phi_f(\omega)} = \sigma_\pm \tag{S10}$$

where $\tilde{\sigma}_\pm(\omega)$ represents the frequency-dependent nonlinear amplitude. We then consider the conservation of energy and momentum

$$2\omega_p - \omega_s = \omega_f \Rightarrow 2k_p - k_s = k_f \tag{S11}$$

and relate the path lengths of the interferometer's arms

$$d' = d - \delta \tag{S12}$$

$$2k_s \delta = \psi. \tag{S13}$$

The parameter δ is the path difference between the interferometer arms that leads to a phase difference of ψ on the seed (idler). Substituting Eqs. (S10-S13) into Eqs. (S6-S9) leads to the equations below.

$$E_{f1-} = \sigma_- \tag{S14}$$

$$E_{f1+} = t_f \sigma_+ e^{2ik_f d} \tag{S15}$$

$$E_{f2-} = t t_s t_p^2 \sigma_- e^{i\gamma} e^{2ik_f d + i\psi} \tag{S16}$$

$$E_{f2+} = t t_f t_s t_p^2 \sigma_+ e^{i\gamma} e^{4ik_f d + i\psi} \tag{S17}$$

Across Eqs. (S14-S17) there are three time/phase windows at which FWM (signal) reaches the detector, separated by the time taken for light to traverse the interferometer 0, $2k_f d$ and $4k_f d$. The coherence time of these temporal windows is limited by the shortest pulse duration in our system (τ_p =~150 fs[1]). As $2d \gg c\tau_p$ and the repetition rate of the laser, 80 MHz, is mismatched with the interferometer arm lengths, ~20 cm, only light arriving within the same temporal window will interfere. Therefore, when calculating the intensity at the detector, D, fields arriving within the same temporal window are grouped together.

$$D = |E_{f1-}|^2 + |E_{f1+} + E_{f2-}|^2 + |E_{f2+} + E_{f1+} r_f e^{2ik_f d}|^2 \tag{S18}$$

An additional term has been included due to the back reflection of E_{f1+} off the metasurface leading to a temporal overlap with E_{f2+} at $4k_f d$. Linear reflections of the pump, seed and FWM will create successive temporal overlap at $mk_f d$, where m is an even integer. However, the nonlinear scaling of FWM emission with pump and probe amplitude allows the model to be approximated to the first FWM reflection. Substituting in the amplitudes in Eqs. (S15-S16) and normalising the detected signal relative to the forward FWM emission signal, $N = D/\sigma_+^2$, gives us

$$N = R_f T_f + (\eta^2 + T_f)(1 + \kappa T) + 2(\eta + \sqrt{T_f R_f})\sqrt{\kappa T T_f}\cos(\gamma + \psi) \tag{S19}$$

where $T = |t|^2$ and $R = |r|^2$, $\kappa = T_p^2 T_s$ is the ratio of total FWM (signal) from the two passes, and $\eta = \sigma_- / \sigma_+$ is the ratio of emission in the backwards and forwards directions. Calculating visibility as $V = \frac{N_{max} - N_{min}}{N_{max} + N_{min}}$ gives

$$V = \frac{2\sqrt{\kappa T T_f}(\eta + \sqrt{T_f R_f})}{R_f T_f + (\eta^2 + T_f)(1 + \kappa T)}. \tag{S20}$$

Supplementary Note III. Mach-Zehnder configuration

In the Michelson style interferometer discussed above and in the main text, one of the four nonlinear fields only contributes to the background, as it is isolated in time from all other fields. However, in a Mach-Zehnder configuration, Supplementary Fig. 2, fields are directionally filtered to improve visibility.

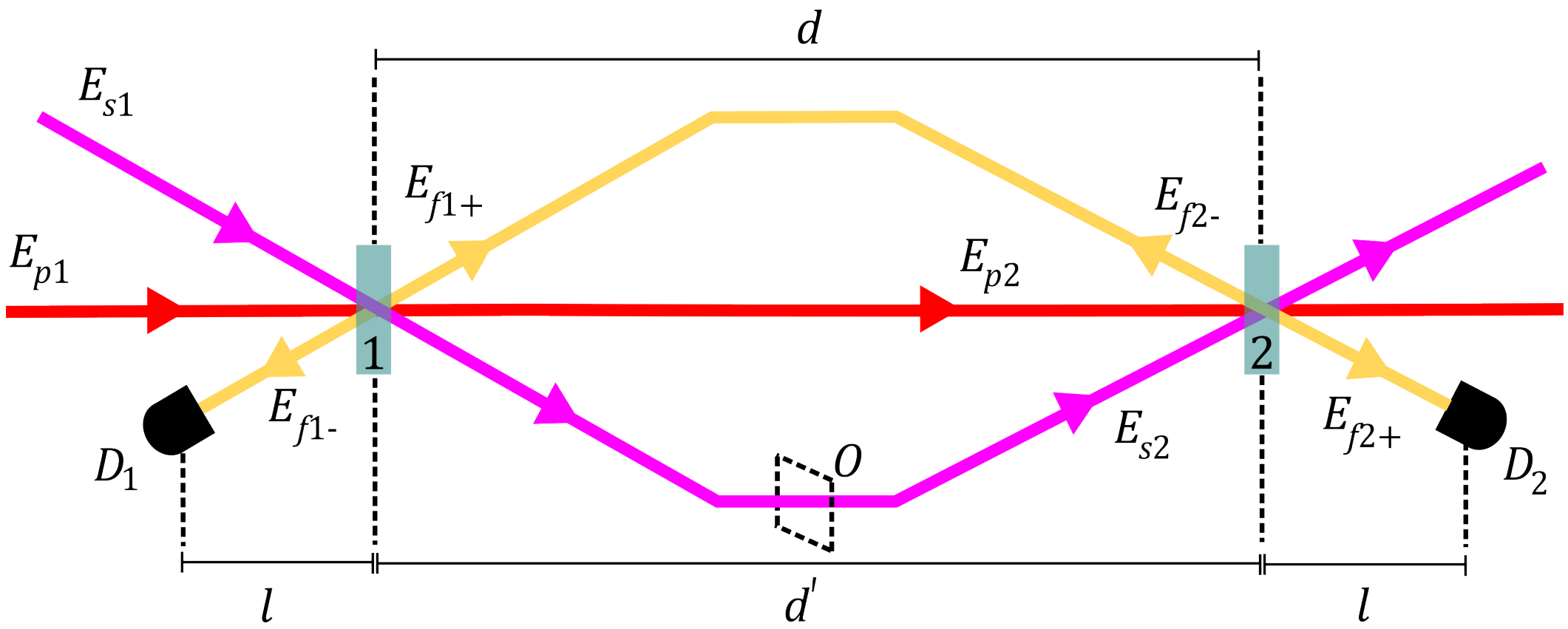

Supplementary Figure 2. A Mach-Zehnder interferometer with two nonlinear metasurface sources. The colours of the paths mark the pump (red), seed (idler, magenta) and FWM (signal, yellow). The fields $E_{f1\pm,f2\pm}$ represent the bidirectional emission at the first and second source, respectively. Detector D_1 measures the backwards emitted FWM fields and detector D_2 measures the forward emitted fields. Inside the interferometer, the FWM and pump travel path d and the seed travels path d'.

In the proposed setup, a pump and seed pass through two identical metasurface or thin-film sources, generating bidirectional emission at each. E_{p1} and E_{s1} are the pump and seed fields at the first source, which generate FWM mixing fields E_{f1+} in the forward direction and E_{f1-} in the backward direction. E_{p2} and E_{s2} are the pump and seed fields at the second source that generate FWM fields E_{f2+} in the forward direction and E_{f2-} in the backward direction. Detector 1, D_1, measures the intensity of the backward-generated fields and detector 2, D_2, measures the intensity of the forward-generated fields.

The incident fields at the first source are defined as in Eqs. (S1) and (S2) in the derivation above.

From the paths labelled in Supplementary Fig. 2, the pump and seed fields at the second source are

$$E_{p2} = t_p E_{p1} e^{ik_p d} \tag{S21}$$

and

$$E_{s2} = t_s t e^{i\gamma} E_{s1} e^{ik_s d'}. \tag{S22}$$

Here, $t_{p,s}$ are the transmission coefficients of the pump and seed beam amplitudes through the first source, and t and γ are the transmission coefficient and phase accrued by the seed (idler) at the object plane O. As we have assumed the two sources are identical, the nonlinear fields generated at each source have the form identified in Eq. (S5). As before, we assume the pump and seed are undepleted by the FWM process. From here, we assess the FWM fields at the respective detectors.

$$E_{f1-} = \alpha_- E_{p1}^2 E_{s1}^* e^{ik_f l} \tag{S23}$$

$$E_{f1+} = \alpha_+ t_f E_{p1}^2 E_{s1}^* e^{ik_f (d+l)} \tag{S24}$$

$$E_{f2-} = \alpha_- t_f E_{p2}^2 E_{s2}^* e^{ik_f (d+l)} = \alpha_- t_f t_s t_p^2 t e^{i\gamma} E_{p1}^2 E_{s1}^* e^{i2dk_p - id'k_s} e^{ik_f (d+l)} \tag{S25}$$

$$E_{f2+} = \alpha_+ E_{p2}^2 E_{s2}^* e^{ik_f l} = \alpha_+ t_s t_p^2 t e^{i\gamma} E_{p1}^2 E_{s1}^* e^{i2dk_p - id'k_s} e^{ik_f l} \tag{S26}$$

The FWM and pump beams take a path of length d and the seed light takes a path of length d', if directed through the interferometer. The conservation of energy and momentum in the FWM process is stated in Eq. (S11) and the path difference within the interferometer is defined in Eq. (S12). This path difference produces a phase shift.

$$k_s \delta = \psi \tag{S27}$$

We also collect like terms into one parameter for each direction, that represents the average nonlinear amplitude in Eq. (S10). These simplifications are substituted into the FWM fields in Eqs. (S23-S26).

$$E_{f1-} = \sigma_- \tag{S28}$$

$$E_{f1+} = \sigma_+ e^{ik_f d} \tag{S29}$$

$$E_{f2-} = \sigma_- t_f t_s t_p^2 t e^{i\gamma} e^{i\psi} e^{i2k_f d} \tag{S30}$$

$$E_{f2+} = \sigma_+ t_s t_p^2 t e^{i\gamma} e^{i\psi} e^{ik_f d} \tag{S31}$$

As before, we have separate temporal/phase windows defined by the paths taken by the various fields. The two forward generated fields overlap at $k_f d$, while the backward generated fields are separated from one another by $2k_f d$. We make the assumption that $d \ll cR^{-1}$, where R is the repetition rate of our laser, such that subsequent pulses do not overlap. However, the E_{f1+} field reflects off source 2 towards detector 1 and is overlapped with the E_{f2-} field. We define the total intensity at D_1.

$$D_1 = |E_{f1-}|^2 + |E_{f2-} + r_f e^{ik_f d} E_{f1+}|^2 \tag{S32}$$

Substituting Eqs. (S28-S30) into Eq. (S32) leads to

$$D_1 = |\sigma_-|^2 + |\sigma_- t_f t_s t_p^2 t e^{i\gamma} e^{i\psi} e^{i2k_f d} + t_f r_f \sigma_+ e^{i2k_f d}|^2. \tag{S33}$$

Expanding Eq. (S33) and normalising to σ_+^2 results in N_1.

$$N_1 = \frac{D_1}{\sigma_+^2} = \eta^2(1 + \kappa T_f T) + T_f R_f + 2\eta T_f \sqrt{\kappa R_f T} \cos(\psi + \gamma) \tag{S34}$$

Here, $T = |t|^2$ and $R = |r|^2$, $\kappa = T_p^2 T_s$ is the ratio of the total FWM (signal) from the two passes, and $\eta = \sigma_- / \sigma_+$ is the ratio of emission in the backward and forward directions. The above equation has oscillating and non-oscillating terms, and visibility can be calculated as $V_1 = \frac{N_1^{max} - N_1^{min}}{N_1^{max} + N_1^{min}}$.

$$V_1 = \frac{2\eta T_f \sqrt{\kappa R_f T}}{\eta^2(1 + \kappa T_f T) + T_f R_f} \tag{S35}$$

At D_2 we have interference between the forward emitted fields.

$$D_2 = |E_{f1+} + E_{f2+}|^2 \tag{S36}$$

Substituting in the equations for the fields in Eqs. (S29) and (S31) leads to

$$D_2 = |\sigma_+ e^{ik_f d} + \sigma_+ t_s t_p^2 t e^{i\gamma} e^{i\psi} e^{ik_f d}|^2. \tag{S37}$$

We expand out the above equation and normalise to the forward emitted nonlinear amplitude.

$$N_2 = \frac{D_2}{\sigma_+} = T_f + \kappa T + 2\sqrt{\kappa T T_f} \cos(\gamma + \psi) \tag{S38}$$

From this equation, the visibility can be calculated from $V_2 = \frac{N_2^{max} - N_2^{min}}{N_2^{max} + N_2^{min}}$.

$$V_2 = \frac{2\sqrt{\kappa T T_f}}{T_f + \kappa T} \tag{S39}$$

We have arrived at a scenario where D_1 measures interference with visibility limited by the non-interfering field, similar to that of the Michelson configuration in the main text, but D_2 measures interference theoretically capable of 100% visibility, similar to conventional nonlinear interferometers. Because the E_{s2} field travels through the object plane to generate E_{f2+}, the visibility in the backward direction contains transmission information about the object at O. It is worth noting that the interfering fields that reach D_1 will also reflect off source 1 and reach D_2, but this should not impact the maximum achievable visibility.

Supplementary Note IV. Tilted thin-film metasurface source

An alternative route to separating the nonlinear fields involves tilting the metasurface source(s). This would be effective in a Michelson or Mach-Zehnder configuration; however, it is described here with reference to the former. The diagrams in Supplementary Fig. 3 show the pump, seed (idler) and FWM (signal) fields at the two passes through a tilted metasurface.

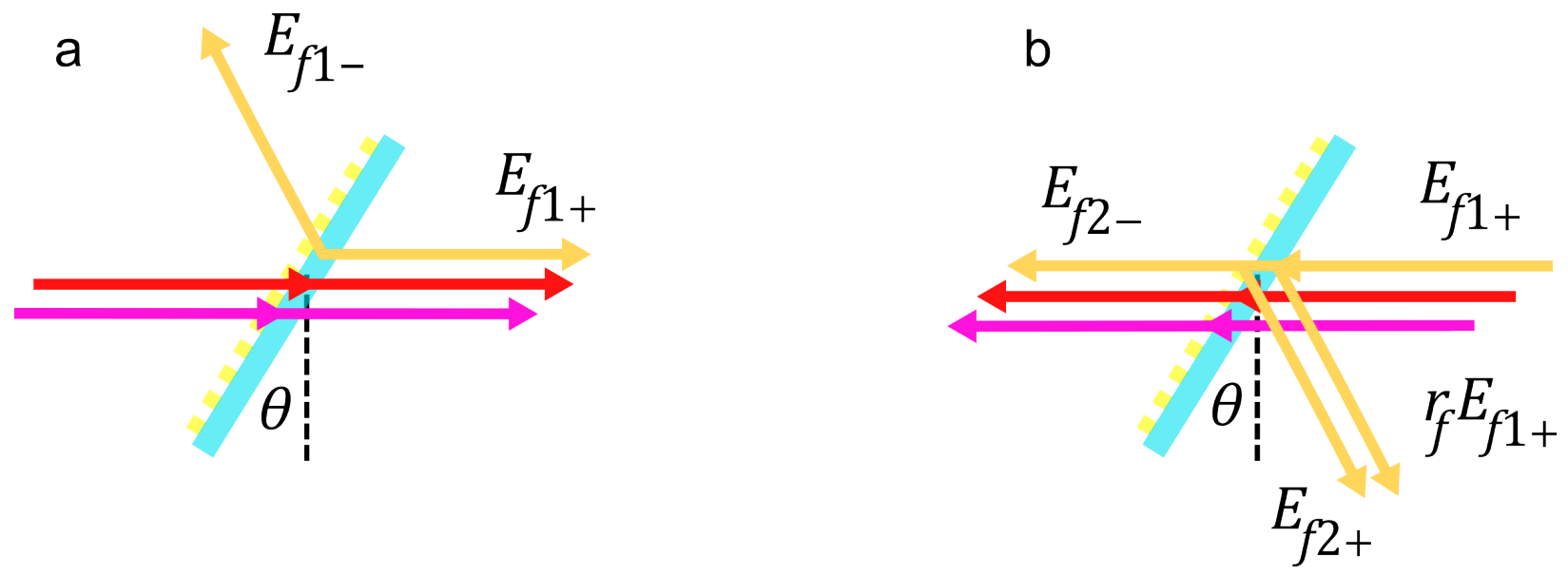

Supplementary Figure 3. Angular misalignment of FWM (signal) fields when the metasurface source is tilted inside a Michelson interferometer. (a) FWM (signal) fields at the first pass, where the E_{f1-} field is reflected away from the optical axis of the pump and seed. (b) FWM (signal) fields at the second pass, where the secondary interference pattern is reflected away from the optical axis.

The metasurface is tilted at an arbitrary angle θ from normal incidence with the optical axis, shared by the collinear pump and seed (idler) beams. At the first pass, the FWM (signal) field generated in the positive direction (E_{f1+}) is emitted along the optical axis. However, the FWM (signal) field generated in the negative direction (E_{f1-}) is emitted $-\theta$ from the sample normal (2θ from the pump-seed optical axis). The emission angle is due to the in-plane momentum conservation of the nonlinear process at the metasurface. A similar scenario is expected at the second pass, where E_{f2-} is emitted along the optical axis, but the E_{f2+} field is emitted at $-\theta$. The first pass field is reflected from the metasurface ($r_f E_{f1+}$) at the same angle. Therefore, the tilted metasurface introduces three paths, one for each of the three temporal/phase windows identified in Supplementary Note II. As each set of fields is now separated angularly as well as by their phase, we define the nonlinear amplitude and FWM (signal) fields without path information.

$$\alpha_\pm E_p^2 E_s^* e^{i(2\phi_p(\omega)-\phi_s(\omega))} = \tilde{\sigma}_\pm e^{i\phi_f(\omega)} = \sigma_\pm \tag{S40}$$

$$E_{f1-} = \sigma_- \tag{S41}$$

$$E_{f1=} = \sigma_+ \tag{S42}$$

$$E_{f2-} = tt_s t_p^2 \sigma_- e^{i\gamma} e^{i\psi} \tag{S43}$$

$$E_{f2+} = tt_s t_p^2 \sigma_+ e^{i\gamma} e^{i\psi} \tag{S44}$$

By placing detectors outside the interferometer to terminate each path, the intensity of each set of fields can be measured separately.

$$D_1 = |E_{f1-}|^2 = \sigma_-^2 \tag{S45}$$

$$D_2 = |t_f E_{f1+} + E_{f2-}|^2 = |\sigma_+ t_f + tt_s t_p^2 \sigma_- e^{i\gamma} e^{i\psi}|^2 \tag{S46}$$

$$N_2 = \frac{D_2}{\sigma_+^2} = T_f + TT_s T_p^2 \eta^2 + 2\eta\sqrt{T_f TT_s T_p^2}\cos(\gamma+\psi) \tag{S47}$$

$$D_3 = |E_{f2+} + r_f E_{f1+}|^2 = |tt_s t_p^2 \sigma_+ e^{i\gamma} e^{i\psi} + \sigma_+ r_f|^2 \tag{S48}$$

$$N_3 = \frac{D_3}{\sigma_+^2} = TT_s T_P^2 + R_f + 2\sqrt{T_s T_P^2 R_f T}\cos(\gamma+\psi) \tag{S49}$$

In the above equations, $T = |t|^2$ and $R = |r|^2$, $\kappa = T_s T_p^2$ and $\eta = \sigma_-/\sigma_+$, as before. It is evident that

D_1 measures a single field and there is no interference. Two fields are expected at both D_2 and D_3, and the normalised intensity at both detectors contain DC and oscillating terms. A theoretical visibility is calculated at each detector using $V = \frac{N_{max}-N_{min}}{N_{max}+N_{min}}$, as applied in earlier derivations.

$$V_2 = \frac{2\eta\sqrt{\kappa T_f T}}{T_f + \kappa\eta^2 T} \tag{S50}$$

$$V_3 = \frac{2\sqrt{\kappa R_f T}}{R_f + \kappa T} \tag{S51}$$

Both V_2 and V_3 are dependent on T and are theoretically capable of reaching 100% visibility, limited only by the optical properties of the applied metasurface source. V_3 is not dependent on η due to both fields being emitted in the positive direction. The non-interfering nonlinear signal that does not reach the object plane can be simultaneously monitored at D_1.

Supplementary Note V. Predicted visibility

The predicted visibility is calculated from images of the non-interfering fields at the camera when a beam block is placed in the respective arms of the interferometer. Supplementary Fig. 4(a) and (b) show these images labelled with the combination of FWM fields and linear optical properties of the metasurface that define them. Far-field images of $\sigma_\pm$ and η calculated from Supplementary Fig. 4(a) and (b) are shown in (c-e). The far-field measurement of η is then used to predict the visibility across the camera using the parameters of the characterised metasurface to give Supplementary Fig. 5(a). The transmission of one pass through the anti-reflection-coated silicon window was measured directly to be 91.3 ± 0.9% at 1500 nm, and was subsequently used to calculate Supplementary Fig. 5(b). Here, the increase in optical path length due to the silicon window, which in turn reduces visibility, has not been considered.

References

[1] Sylvain D. Gennaro, Yi Li, Stefan A. Maier, and Rupert F. Oulton. Double blind ultrafast pulse characterization by mixed frequency generation in a gold antenna. *ACS Photonics*, 5(8):3166–3171, 2018. doi: 10.1021/acsphotonics.8b00387.

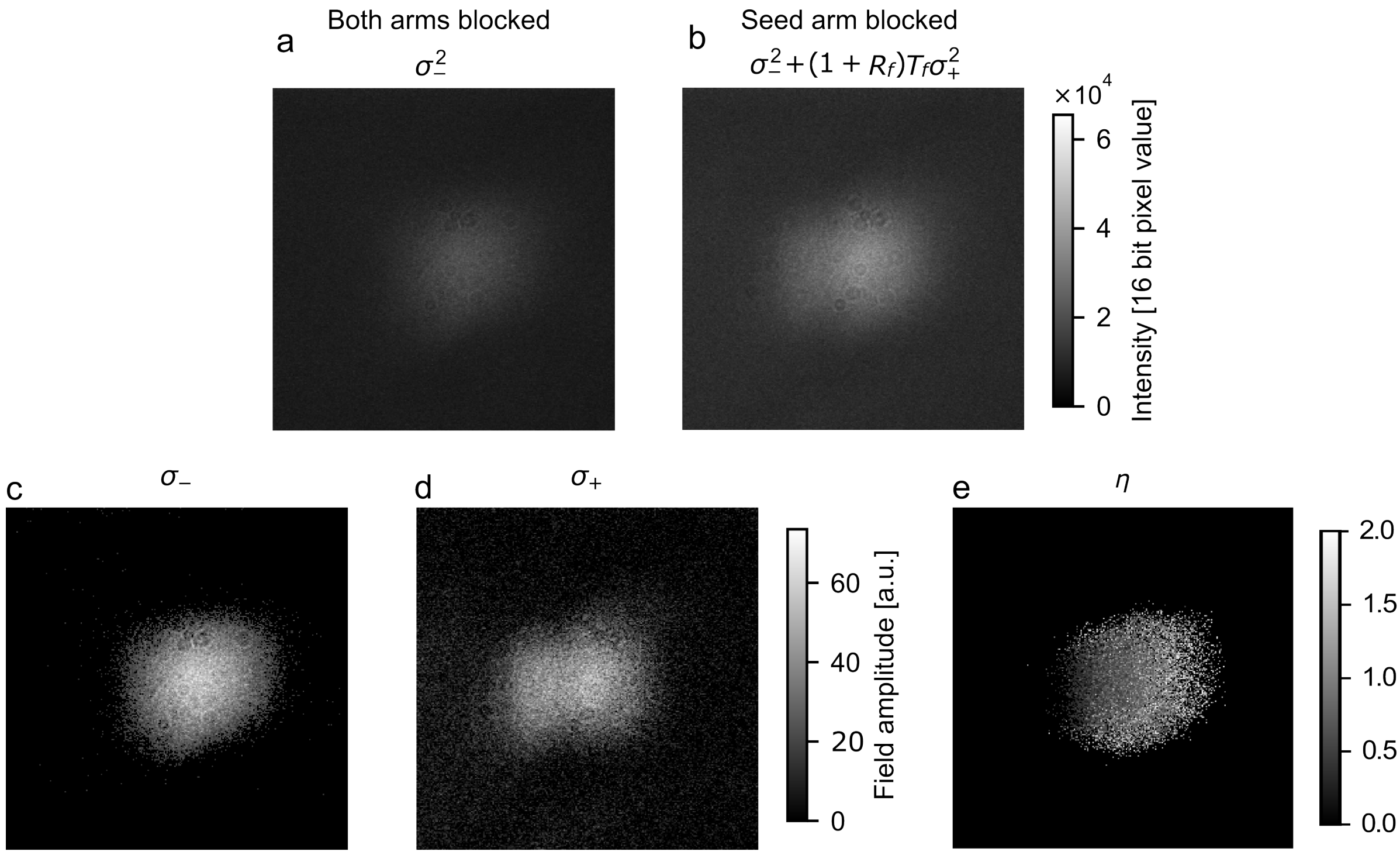

Supplementary Figure 4. Extracted amplitudes of bidirectional FWM emission and η. (a) Camera response to only first pass reflected emission, E_{f1-}. (b) Camera response to the reflected, E_{f1-}, and transmitted, E_{f1+}, first pass emission. Amplitude of FWM emission at the metasurface in the (c) − and (d) + direction. (e) η calculated from (c) and (d).

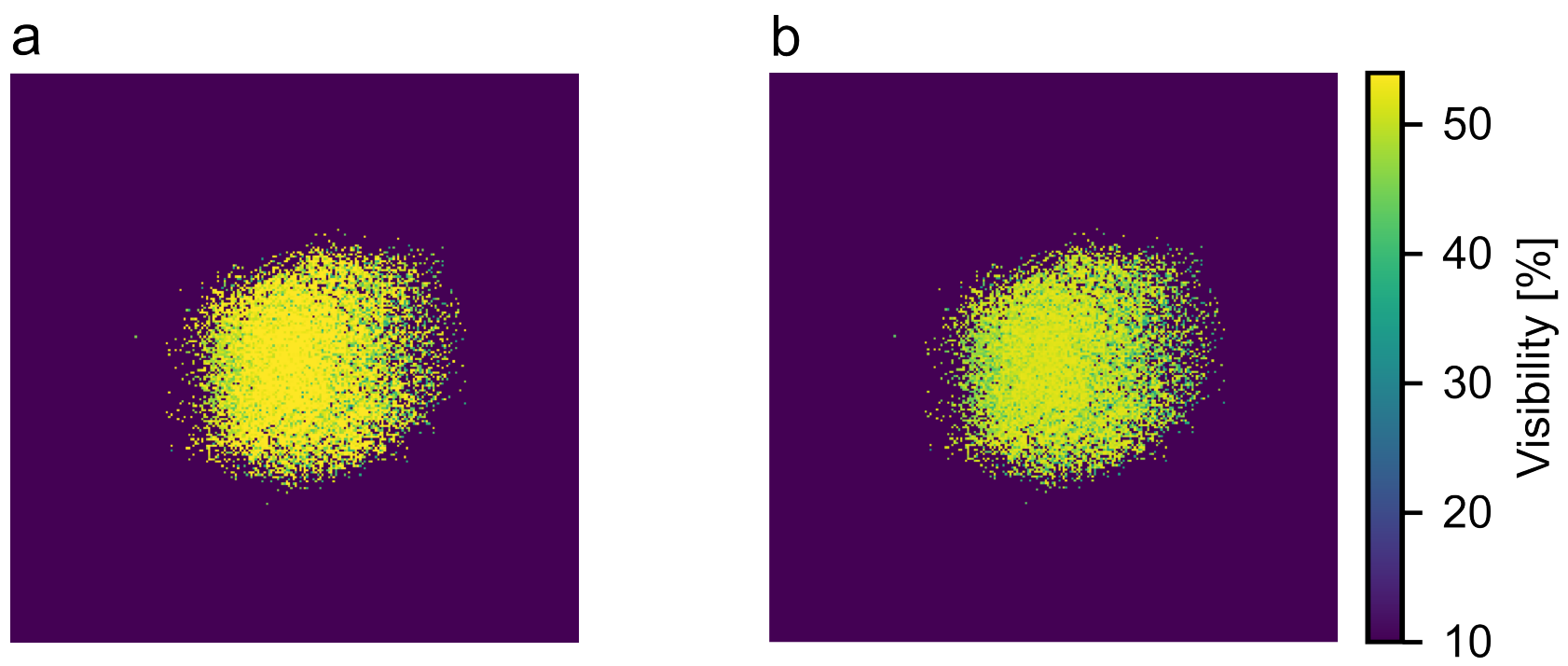

Supplementary Figure 5. Expected visibility from extracted η and linear optical properties of the metasurface in Supplementary Table I. (a) Calculated visibility when T=100%. (b) Calculated visibility when T=83.3±1.6%; the measured transmission of two passes through the 240 µm thick silicon window at 1500 nm.
