# Supplementary material for: Phase-matching-free sensing with undetected light using a nonlinear thin-film metasurface
Source: Nat Commun. 2026 Aug 22;17:10117. doi: 10.1038/s41467-026-76512-5 (PMC13598124; doi:10.1038/s41467-026-76512-5)
Supplement: Supplementary file 2 [file 41467_2026_76512_MOESM2_ESM.pdf]

## Description of Additional Supplementary Files

**Supplementary Video 1.** Four-wave mixing (signal) interference captured on the silicon camera as the phase is varied by translating the piezo-coupled mirror inside the interferometer (curved mirror 1) along the optic axis.
