# Supplementary material for: Phase-matching-free sensing with undetected light using a nonlinear thin-film metasurface
Source: Nat Commun. 2026 Aug 22;17:10117. doi: 10.1038/s41467-026-76512-5 (PMC13598124; doi:10.1038/s41467-026-76512-5)
Supplement: Supplementary file 3 [file 41467_2026_76512_MOESM4_ESM.pdf]

nature portfolio

Peer Review File

Phase-Matching-Free Sensing with Undetected Light Using a Nonlinear Thin-Film Metasurface

Corresponding Author: Dr Toby Severs Millard

This file contains all reviewer reports in order by version, followed by all author rebuttals in order by version.

Version 0:

Reviewer comments:

Reviewer #1

(Remarks to the Author)
In their work, T. S. Millard et al. implement a nonlinear Michelson-type interferometer based on seeded four-wave mixing (FWM) in a plasmonic nonlinear metasurface (MS). Such interferometers are popular now due to their use for 'undetected photons' imaging, spectroscopy, and OCT. In the current experiment, however, spectroscopy or OCT are impossible because FWM is seeded and therefore narrowband; meanwhile, imaging is impossible too because seeding is single-mode. But, to the best of my knowledge, this is the first experiment where a metasurface is used in a nonlinear interferometer. The work is therefore novel and interesting as another application of metasurfaces in quantum (or, rather, quantum-inspired) photonics.

In the experiment, a fs pulsed pump in the NIR range (835 nm) and a fs pulsed seed at a Telecom wavelength are fed into the MS and generate visible (580 nm) FWM signal, which is detected at the output of the nonlinear interferometer with a silicon camera. The MS has been studied in earlier papers by the same authors, in terms of both linear and nonlinear properties. In this experiment, it is resonant for both the pump and the seed, which leads to the enhancement of FWM efficiency.
The authors present a detailed theory of nonlinear interference occurring in this experimental setting, which is rather complicated because the metasurface emits radiation in both forward and backward directions. The visibility is reduced, compared to the case of a bulk FWM or parametric down-conversion source. The authors also test how the interference changes as an object is placed into the Telecom arm of the interferometer. They vary the transmission by covering part of the beam with a knife edge and see how the visibility changes. This shows the potential of using the MS-based interferometer to measure the transmissivity of an object at an undetected (Telecom, in this case) wavelength.

The advantage of using a MS, as we can understand from the title, is the absence of phase matching constraint. In non-resonant sources of FWM, it would be a great advantage because it would enable the use of any wavelength for the seed beam. But in this MS, the seed beam wavelength has to coincide with the resonance. This eliminates the advantage. It looks like the MS is not suitable as a nonlinear source for such interferometry. For this reason, I do not see this work as a major advancement in the field – neither in sensing with undetected photons nor in nanophotonics. I do not therefore recommend publication in Nature Communications; however, the work will be suitable for a more specialized journal like Optics Letters or Optics Express.

There are also minor flaws that should be considered:
1. Page 4: alpha_+ and - characterize the FWM forward and backward efficiencies. Their ratio is important for the theoretical description of the nonlinear interference. But the paper gives no considerations about the relation between them. I believe such considerations can be derived from the MS structure?
2. Fig.2: the caption says it shows the visibility but instead, it shows the interference pattern.
3. In the description of this figure, the drop of visibility is mentioned as 24.4%+-12.2%. This is a very uncertain estimate; why?
4. Caption to Fig.3: 'The experimentally measured and optimized values are marked by the vertical lines.' I cannot see how vertical lines mark any values.
5. Further in the description: the visibility is normalized, but it is not clear how it is normalized: to the theoretical maximum? Or to the experimental maximum?
6. In Fig.4, the fine modulation is explained by the Fabry-Perot effect. But I doubt it is possible with pulses shorter than 200 fs

in a 175-micrometer thick slab.

Reviewer #2

(Remarks to the Author)
The paper entitled "Phase Matching Free Sensing with Undetected Light Using a Nonlinear Metasurface" by Millar et al, demonstrates a method of classical sensing with undetected light using stimulated four-wave mixing (FWM) and a gold plasmonic nonlinear metasurface. The metasurface advantage enables phase-matching-free sensing, transferring amplitude and phase information from near-infrared (NIR) light (1500 nm) to visible light (580 nm). A Michelson-style interferometer is used, achieving over 50% visibility despite inherent reflections. Experiments include sensing through opaque silicon and modulating single-mode transmission with a knife edge. The spectral resolution reveals dispersion effects, enabling ultrafast sensing applications. The metasurface's subwavelength thickness and resonant properties eliminate phase-matching constraints, offering potential for sensing in the mid-infrared (MIR) range and ultrafast spectroscopy.

The work is of significant interest and importance to the field, with the advent of metasurfaces being more readily used in sensing applications and for developing FWM methodologies without bulk nonlinear crystals. The work has sound analysis and interpretations, and provides sufficient detail for reproduction and theoretical understanding.

Some issues that I feel need rectifying are highlighted below:

1) It seems a little distracting on Page 2 in the introduction section, that the 2nd paragraph finishes with the statement of classical sensing being advantageous versus quantum sensing, but then paragraph 3 goes on to discuss the achievement of quantum sensing, before chapter 4 then goes back onto the topic of classical sensing being uses here. It's somewhat dissonant, so I think rewording these paragraphs to make it especially clear that you are not looking at a quantum sensing mechanism is needed.

2) Equation 1 would benefit with an additional =..... of the terms with αEpEs added to the right of the σ.

3) Could the authors add in the corresponding wavelengths as insets in to the subfigures for figure 2, this makes it easier to understand the visibility percentages when mentioning the different wavelengths of the pump and FWM wavelengths.

4) Line 198 states "Interestingly, a Fourier transform of the interferometer visibility and phase
199 would reveal ultrafast transients of the object transmission with a resolution less than the pulse duration." The language here implies that it "would" reveal features but hasn't been done or shown in the manuscript. Could the authors comment on this, and if so include a FT plot highlighting these features?

Reviewer #3

(Remarks to the Author)
The authors developed a nonlinear interferometer utilizing a metasurface as nonlinear media and demonstrated sensing with undetected photons by exploiting stimulated four-wave mixing within the metasurface. Using the system, they measured the interference at the interferometer output and evaluated the visibility and phase. They also discussed the effect of bidirectional FWM emission and the existence of the Si sample on the visibility. While a paper already exists that uses a metasurface in the sample arm of the interferometer [Paterova et al. Nanophotonics, 10 1775(2021), applying a metasurface as nonlinear media for the nonlinear interferometer is novel and conceptually interesting.

However, this paper insufficiently shows how a metasurface can benefit undetected photons techniques and improve their performance. The results do not show or discuss how the unique characteristics of a metasurface, bidirectional emission or phase-matching free features, which are not present in conventional nonlinear media, are beneficial for sensing with undetected photons. The data presented as proof of principle is also insufficient to demonstrate the potential of this method as a future imaging or spectroscopy technique. Therefore, unfortunately, I cannot recommend this manuscript for publication in Nature Communications. The detailed points are as follows:

・ Metasurfaces can exhibit resonance over a wide wavelength or wavevector range due to their phase-matching-free feature, which benefits imaging or spectroscopy applications. However, as the authors mention, the seed pulse restricts the FWM signal to a single mode, offsetting the advantages.

・ I understand that a femtosecond seed pulse improves the brightness of the FWM signal and allows sensing with undetected photons in a realistic detection photon number and measurement time. In this case, this method needs to be compared to conventional femtosecond upconversion sensing [e.g., Ashik A. S. et al. Photonics Research 7 783 (2019)., Johnson and Diddams, Appl. Phys. B 107 31(2012)], in my opinion. What is the merit of using this seeded FWM interferometer against them? If a femtosecond light source is eventually required, one of the advantages of undetected photons methods, which is simple with a monochromatic pump source and a nonlinear medium, will be lost, and it will be no different from upconversion techniques. Additionally, the true value of undetected photon techniques is demonstrated in the mid-infrared or ultraviolet region; however, this method requires mid-infrared femtosecond seed pulses, which is a similar situation to that of upconversion techniques.

・ The authors mention the potential for ultrafast measurements using the femtosecond seed pulses, but this is not a unique characteristic of this method. Some researchers have already demonstrated sensing with undetected photons using pulsed pump, signal, and idler [Kaur et al. Phys. Rev. Applied 22, 044015 (2024), Hashimoto et al. Commun. Phys. 7, 217 (2024)]. The pulse durations are picoseconds, but the hundreds of femtoseconds can be achieved by reducing the crystal length.

・ The bidirectional FWM emission is a unique and interesting feature of using a metasurface. The authors demonstrate that directivity results in reduced visibility compared to the ideal case when utilizing a conventional nonlinear medium, which is a disadvantage of using a metasurface. Are there any significant advantages of the bidirectional emission technique over the undetected photons techniques, such as simultaneous sample and background measurement? I suppose there would be a unique advantage to using a metasurface over conventional crystal-based interferometers, and I believe the advantage should be highlighted.

・ Their developed system, consisting of two curved mirrors with a Si sample in the seed arm, insufficiently shows the potential for future spectroscopy or imaging applications. The visibility drop shown in Figure 2 is not mainly associated with the Si's transmittance but rather with the change in beam divergence after adjusting the temporal delay, in my understanding. The phase change is also not directly related to the absolute Si phase. Although this method operates in a single-mode regime, it may be necessary to collimate the seed beam with a lens (and focus) and irradiate it onto a sample, assuming imaging/spectroscopy applications.

【Minor points】
・I suggest that the authors describe the source and detector parameters, including the pump and seed powers (or pulse energy), the generated FWM signal power, the detected signal power in the camera, and the exposure time/ measurement time of the detector. The information may help readers understand that the brightness and signal-to-noise ratio are improved by the seeding effect.

・I guess that the authors describe the equations assuming that the spontaneous emission caused only by the pump is small enough and the related quantum interference is negligible. If it is so, I recommend that the authors mention it.

・I am unsure of "homodyne advantage" in this context, written in Line 56. Do the authors mean the nonlinearity of the visibility vs. transmissivity due to the existence of the seed pulse?

・I am unsure of "signal interference independent of pump-seed delay" in Line 57, and it is not discussed in detail in the latter part. The interferometric visibility depends on the coherence time of the pump. If the authors meant that the interference range can only be determined by the idler optical path-length difference in the time-domain case, it has already been discussed in the previous work [Kaur et al. Phys. Rev. Applied 22, 044015 (2024)].

・A signal pulse can be generated when the pump and seed beams are reflected off the metasurface, enter the interferometer again, and then hit the metasurface again. Is the signal weak enough to be ignored?

・I recommend that the authors add scale bars to the images in Figures 1 (f-h) and 2.

・Why does the interferometric visibility in Figures 2(a) and (b), especially the high-visibility part in the center, have a non-uniform distribution?

・I suggest that the authors explain the details of the axes in Figure 3(e). I am unsure of where the knife-edge position is, the meaning of 0.00 mm in this context, and the meaning of X-axis. Is the X-axis image size on the camera?

Version 1:

Reviewer comments:

Reviewer #1

(Remarks to the Author)
In the rebuttal letter, Millard et al. respond to my main comment, concerning the usefulness of stimulated FWM in their plasmonic metasurface for sensing with undetected photons. While the potential of this work to be impactful for quantum sensing is arguable, I agree that the observation of interference in an SU(1,1) interferometer based on a metasurface is indeed a breakthrough. Therefore, I am, in principle, not objecting against the publication in Nature Communications.
But I still have doubts about certain technical points.

1. To my previous comment about Fig.2, the authors insist that panels a,b show the visibility and not the interference pattern, as it looks like. But then, why does the visibility oscillate approximately the same way as the cos(phi) function? It is supposed to be the factor in front of the cosine, as described in the paragraph above the figure.

2. I did not notice it before, but the abstract says: '…we report classical sensing with undetected light using octave spanning stimulated four-wave mixing from a plasmonic metasurface.' I do not see any octave-spanning spectrum here.

3. In the recent paper Son et al., Opt. Lett. 50, 4434 (2025), it is shown that FWM from a substrate can compete with the FWM

from a strongly nonlinear film. Did the authors check that their substrate does not contribute to the effect?

Reviewer #3

(Remarks to the Author)
I appreciate the authors' careful efforts to address all of my comments, both major and minor, and I believe that the revisions have significantly improved the manuscript. However, I remain unconvinced that one of my main concerns has been adequately addressed, namely, whether this proof-of-principle experiment demonstrates the capability for sensing with undetected photons.

The revised manuscript provides a thorough and well-written discussion of the characteristics of metasurface-based nonlinear interferometry, including its theoretical foundations. It explains that a Mach-Zehnder configuration can achieve near-100% visibility, and that a similar improvement can be obtained by tilting the metasurface. The tilted geometry further enables simultaneous detection of nonlinear emission, which is particularly interesting, as it may offer capabilities not available in conventional approaches. In addition, the authors emphasize the advantages of metasurfaces, including the ability to engineer the wavelength, bandwidth, and spatial mode of biphotons without being constrained by phase-matching conditions, thereby further characterizing this platform. The ease of maintaining transform-limited pulses is also noteworthy and may indeed provide advantages over conventional methods. Overall, these discussions are highly informative and strengthen the manuscript.

Nevertheless, I still do not think that the presented proof-of-principle experiment convincingly demonstrates applicability to sensing with undetected photons. First, the results shown in Fig. 2 appear to primarily confirm the basic operation of the undetected-photons scheme, rather than demonstrating sensing. Although the authors state that "in Fig. 4 the technique is applied to spectroscopy" and that "the application to spectroscopy was demonstrated in Fig. 4," the experiment in Fig. 4 essentially extracts the fine spectral fringe arising from an etalon effect generated when the pump propagates through a borosilicate substrate. While frequency-resolved measurements are indeed performed, they do not constitute genuine spectroscopic sensing of a sample, such as the detection of idler absorption or phase shifts induced by a target object, nor are the results quantitatively evaluated or compared with known reference values. Although the spectrogram is useful for assessing dispersion within the system, it does not directly evaluate an object's sensing capability in the context of the undetected photons method.

Conversely, if the main goal is to emphasize characterization of the nonlinear interferometer itself rather than validation of sensing, then it would be important to experimentally verify the distinctive features of a metasurface-based nonlinear interferometer (aside from the side effect of bidirectional photon emission). At present, many of these features remain at the level of theoretical analysis and prediction. Taken together, the current proof-of-principle does not go beyond demonstrating the simple combination of a metasurface and a nonlinear interferometer.

【Other comments】
I recommend that the authors further clarify the four advantages they claim relative to the upconversion approach. At least in upconversion interferometry (e.g., optical coherence tomography), where the infrared light is sent through an interferometer and then upconverted, the (relative) phase of the sample can be measured and evaluated. If your argument is that interferometric measurement itself is what yields four advantages, then wouldn't an upconversion interferometer achieve essentially the same benefits as well?

Version 2:

Reviewer comments:

Reviewer #1

(Remarks to the Author)
The rebuttal letter by Millard et al. provides a convincing response to my first comment (about the visibility).

As to my second comment, about 'octave-spanning four-wave mixing', the authors still insist on the wording. The text now runs as "…we report octave-spanning classical sensing with undetected light using stimulated four-wave mixing from a plasmonic metasurface." They claim that 'as the sensing wavelength is 1500 nm and the detection wavelength is 580 nm, this spans almost two octaves and therefore can be described as octave spanning.'

I strongly disagree with this formulation. 'Spanning' means covering a range of frequencies. The fact that the detection wavelength and the sensing wavelength are much separated in spectrum is common for all 'undetected photons' techniques; however, none of the works speaks of 'octave-spanning sensing'. This is a misleading statement. The authors should choose a more careful way to indicate the large separation between the two wavelengths.

As to the response to my third comment (about the effect of the substrate), I am fully satisfied.

Reviewer #3

(Remarks to the Author)
I appreciate that the authors addressed as many of my concerns as possible. I already understand what is done in Figures 1-4, and from the response letter I also understand the authors' claim that observing changes in the amplitude or phase of light constitutes sensing in a broad sense; therefore, their verification is consistent with that claim. On the other hand, as I have mentioned in previous review comments, this paper does not actually acquire and evaluate images of objects with a two-dimensional distribution to demonstrate its applicability as an imaging technique, nor does it acquire and evaluate spectra of objects with different absorption/reflection for each frequency to demonstrate its applicability as a spectroscopic technique. Given that many other undetected-photons techniques acquire and evaluate images, spectra, phase images, or tomographic images of objects, I'm still not entirely convinced that this paper has reached the level required for publication in Nature Communications. This is because, within the scope of current verification, it is unclear which applications, including the aforementioned imaging and spectroscopy, this alone would be useful for. On the other hand, I understand their position that, because nonlinear interferometry using metasurfaces differs substantially from other methods, they should limit the verification here to what is minimally necessary for an undetected-photons technique, and that the aforementioned imaging or spectroscopy demonstrations should be the next step in a separate paper. I would like to leave the final decision on publication to the editor, taking these points into consideration.

Open Access This Peer Review File is licensed under a Creative Commons Attribution 4.0 International License, which permits use, sharing, adaptation, distribution and reproduction in any medium or format, as long as you give appropriate credit to the original author(s) and the source, provide a link to the Creative Commons license, and indicate if changes were made.
In cases where reviewers are anonymous, credit should be given to 'Anonymous Referee' and the source.
The images or other third party material in this Peer Review File are included in the article's Creative Commons license, unless indicated otherwise in a credit line to the material. If material is not included in the article's Creative Commons license and your intended use is not permitted by statutory regulation or exceeds the permitted use, you will need to obtain permission directly from the copyright holder.
To view a copy of this license, visit https://creativecommons.org/licenses/by/4.0/

Response to reviewers: Phase Matching Free Sensing with Undetected Light Using a Nonlinear Thin-Film Metasurface – Severs Millard et al

Reviewer #1:

1.1. *In their work, T. S. Millard et al. implement a nonlinear Michelson-type interferometer based on seeded four-wave mixing (FWM) in a plasmonic nonlinear metasurface (MS). Such interferometers are popular now due to their use for 'undetected photons' imaging, spectroscopy, and OCT. In the current experiment, however, spectroscopy or OCT are impossible because FWM is seeded and therefore narrowband; meanwhile, imaging is impossible too because seeding is single-mode. But, to the best of my knowledge, this is the first experiment where a metasurface is used in a nonlinear interferometer. The work is therefore novel and interesting as another application of metasurfaces in quantum (or, rather, quantum-inspired) photonics.*

We would like to thank the reviewer for their positive feedback on this work. It is true that the signal emission and subsequent interference is defined by the spectral and spatial modes of the pump and seed. This is a feature of using stimulated nonlinear emission. It is worth noting that using a seed beam with the desired characteristics, such as spectrally broadband and spatially multi-mode, would allow our system to be better suited for the applications stated, without changing the inherent operation of the interferometer as presented in our work. In the current demonstration, since ultrafast excitation and seed are utilized, the spectral bandwidth in the IR is > 20 nm, which would enable OCT depth resolution ~ 50 um. These values could be improved by using shorter pulse lasers.

1.2. *In the experiment, a fs pulsed pump in the NIR range (835 nm) and a fs pulsed seed at a Telecom wavelength are fed into the MS and generate visible (580 nm) FWM signal, which is detected at the output of the nonlinear interferometer with a silicon camera. The MS has been studied in earlier papers by the same authors, in terms of both linear and nonlinear properties. In this experiment, it is resonant for both the pump and the seed, which leads to the enhancement of FWM efficiency.*

The authors present a detailed theory of nonlinear interference occurring in this experimental setting, which is rather complicated because the metasurface emits radiation in both forward and backward directions. The visibility is reduced, compared to the case of a bulk FWM or parametric down-conversion source. The authors also test how the interference changes as an object is placed into the Telecom arm of the interferometer. They vary the transmission by covering part of the beam with a knife edge and see how the visibility changes. This shows the potential of using the MS-based interferometer to measure the transmissivity of an object at an undetected (Telecom, in this case) wavelength.

This is an accurate summary of the experiment presented. An important point to make is that although the theoretical maximum visibility falls due to non-interfering back reflections, the experimental visibility matches this maximum. In conventional nonlinear interferometry using bulk nonlinear crystals the theoretical visibility is 100%. However, this is often observed to be much lower experimentally. Additionally, if the nonlinear reflections from the metasurface can be detected separately, e.g. using time gating or alternative interferometer configurations, the visibility will rival conventional systems. In section SIII of the SI an alternative Mach-Zehnder system is proposed that filters forwards and backwards emitted fields and could, in principle, reach a maximum visibility of 100%, and in section SIV of the SI 100% visibility is theoretically achieved by tilting the thin-film metasurface source inside a Michelson interferometer.

1.3. *The advantage of using a MS, as we can understand from the title, is the absence of phase matching constraint. In non-resonant sources of FWM, it would be a great advantage because it would enable the use of any wavelength for the seed beam. But in this MS, the seed beam wavelength has to coincide with the resonance. This eliminates the advantage. It looks like the MS is not suitable as a nonlinear source for such interferometry. For this reason, I do not see this work as a major advancement in the field – neither in sensing with undetected photons nor in nanophotonics. I do not therefore recommend publication in Nature Communications; however, the work will be suitable for a more specialized journal like Optics Letters or Optics Express.*

There are a few points to make clear in response to these valid concerns. Firstly, the plasmonic resonance is characteristically broad and a wide degree of tuning can be achieved by varying the pump and seed wavelengths across this resonance, as is shown by Yang et al. (DOI: 10.1021/acsphotonics.5c00764). The degree of tuning is substantially more than can be achieved with a single conventional nonlinear crystal. Secondly, broadband seeding could fully utilise the broadband plasmonic resonance for spectroscopic applications. By broadband – we imply perhaps > 100 nm. Thirdly, metasurfaces can be custom made with specific resonances for a given application and therefore do enable the use of any seed beam wavelength, at the point of fabrication. In principle, this allows the spectral range of the technique to extend past what is achievable with current bulk nonlinear crystals due to the phase matching free nature of metasurface sources.

Finally, our findings are not only of interest to the application of metasurfaces as nonlinear sources, but to any thin-film source used for nonlinear interferometry, including non-resonant thin-films of nonlinear material. Indeed, nonlinear interference has not be shown even with thin nonlinear optical materials. The work is therefore valuable to the field of nonlinear interferometry and nanophotonics more generally than the reviewer implies, highlighting the operation of the system independently of the thin film nonlinear source applied.

To clarify that metasurface sources offer phase-matching-free nonlinear emission alongside broad spectral emission lines 237-239 now read:

"Similarly, for spectroscopy, the system can utilise the broad spectral range of the resonant metasurface, either by tuning the seeding wavelength or illuminating with a broadband seed beam."

To highlight the invariance of the interferometer model to the thin-film nonlinear source used lines 249-252 have been added:

"Additionally, the interferometer model developed is independent of the thin-film source used, including thin films of nonresonant nonlinear crystals, allowing the operating wavelengths to be defined solely by the pump and seed beams."

1.4. *There are also minor flaws that should be considered:*

4.1 Page 4: alpha_+ and - characterize the FWM forward and backward efficiencies. Their ratio is important for the theoretical description of the nonlinear interference. But the paper gives no considerations about the relation between them. I believe such considerations can be derived from the MS structure?

Yes, FWM is emitted in two directions due to the MS structure. We have characterised the ratio of this emission, $\eta = \frac{\sigma_-}{\sigma_+} = \frac{\alpha_-}{\alpha_+}$, by measuring σ_+ and σ_- at the camera (Fig. S4). We have not considered the theoretical mechanism for the measured ratio of the applied metasurface, $\eta = 0.885$, which would involve in-depth simulation and calculation of the spontaneous and stimulated emission rates in each direction. While the parameter η is important for optimising interference, the submitted article focuses on the operation of nonlinear interferometry with a thin-film metasurface source and not experimental optimisation of the metasurface itself.

1.4.2 Fig.2: the caption says it shows the visibility but instead, it shows the interference pattern.

Fig.2 is showing the visibility and phase, extracted from the interference, with and without a silicon window at the object plane. The fringes observed in the visibility are due to phases of the two interference patterns not being coherent across the camera's field of view. As the system is single mode, only the maximum visibility where the interference patterns coherently sum at the detector is of interest.

1.4.3 In the description of this figure, the drop of visibility is mentioned as 24.4%+-12.2%. This is a very uncertain estimate; why?

Thank you for pointing out this large uncertainty, which was calculated incorrectly. We have recalculated the uncertainty from the covariance matrix of the sinusoidal fit use to extract the visibility from the interference pattern at each pixel. The uncertainties in the visibility before and after the silicon window are found to be

smaller than originally stated, the correct values are 55.0 +/- 1.7% without silicon at the object plane and 24.4 +/- 1.7% with the silicon window at the object plane.

Changes:

Lines 148-149: "We compare the theoretical prediction to the experimental visibility in Fig.2(a) with a maximum of 55.0% ± 1.7%"

Lines 161-162: "...the visibility has dropped to a maximum of 24.4 ± 1.7%."

1.4.4. Caption to Fig.3: 'The experimentally measured and optimized values are marked by the vertical lines.' I cannot see how vertical lines mark any values.

Fig. 3(a-c) shows how the visibility, calculated from Eq. (7), changes with the optical properties of the metasurface, η, T_f and κ. The solid vertical lines on each panel in Fig. 3(a-c) mark the experimentally measured value on the x-axis for the metasurface shown in Fig. 1(b), for the given optical property that is being varied. The dashed vertical lines mark the optical property value that would maximise the visibility, representing the ideal (optimised) metasurface. Fig. 3(d) shows the visibility curve as a function of object plane transmission, where the solid curve represents the theoretical model with the experimentally measured optical properties applied and the dashed line represents the theoretical model with the optimal optical properties applied.

We believe the confusion has come about due to the reference to vertical lines being after the introduction of panel (d), which does not have any vertical lines marked. The figure caption has been rewritten to only refer to vertical lines regarding Fig. 3(a-c), and now reads:

"Theoretical dependence of visibility on (a) η, (b) T_f and (c) κ. The experimentally measured and theoretically optimised metasurface parameters are marked by the solid and dashed vertical lines, respectively. Constant parameters are held at the values in Table SI of the SI. (d) Dependence of visibility on seed (idler) transmission, T, for the experimental measured (solid curve) and theoretically optimised (dashed curve) metasurface parameters."

1.4.5. Further in the description: the visibility is normalized, but it is not clear how it is normalized: to the theoretical maximum? Or to the experimental maximum?

Because the visibility is averaged across the whole of the camera's sensor it is expectedly small and we have therefore normalised the data to the average of the unobstructed measurement, when the knife edge is at zero.

The section of the figure caption has been rewritten and now reads:

"(f) Experimental visibility as the knife edge is introduced, averaged across all pixels and normalised to the unobstructed visibility."

1.4.6. In Fig.4, the fine modulation is explained by the Fabry-Perot effect. But I doubt it is possible with pulses shorter than 200 fs in a 175-micrometer thick slab.

In free space, without the Fabry-Perot cavity (the substrate), the pump laser (835 nm) has individual pulses of ~200 fs duration in the time domain, and a pulse train with a repetition rate 80 MHz. In the frequency domain this appears as a ~8 nm FWHM continuous band due to the resolution of our spectrometer, but is actually a fine comb of very narrow but finite linewidth modes that interfere to produce a pulse train, where the repetition rate is defined by the spacing of the discrete modes and the pulse duration is governed by the spectral shape and range of the combined modes. The Fabry-Perot cavity acts as a filter, selecting out frequencies with a free spectral range $\Delta\nu_{FSR} = \frac{c}{2Ln}$, where L is the cavity length and n is the refractive index. Although in time it would appear the pulses cannot overlap due to the cavity, if we remain in the frequency domain it can be modelled quite simply. We take the intensity of a single pulse

$$\psi(\lambda) = e^{-\frac{(\lambda-\lambda_0)^2}{2\sigma^2}}.$$

Here, λ_0 is the central wavelength and σ is the standard deviation (width). The transmission of a Fabry-Perot cavity can be defined as

$$T(\lambda) = \frac{1}{1 + \frac{4F^2}{\pi^2} \sin\left(\frac{\phi(\lambda)}{2}\right)^2}.$$

The finesse of the cavity is $F = \frac{\pi \sqrt[4]{R_1 R_2}}{1-\sqrt{R_1 R_2}}$ where R_1 and R_2 are the reflections at each interface of the cavity and the phase shift across the cavity is $\phi(\lambda) = \frac{4\pi n L}{\lambda}$. Assuming no absorption and an equal reflection at each interface of 0.06, the transmitted $\psi(\lambda)T(\lambda)$ and reflected $\psi(\lambda)\left(1 - T(\lambda)\right)$ intensities are shown in Fig. R1(a).

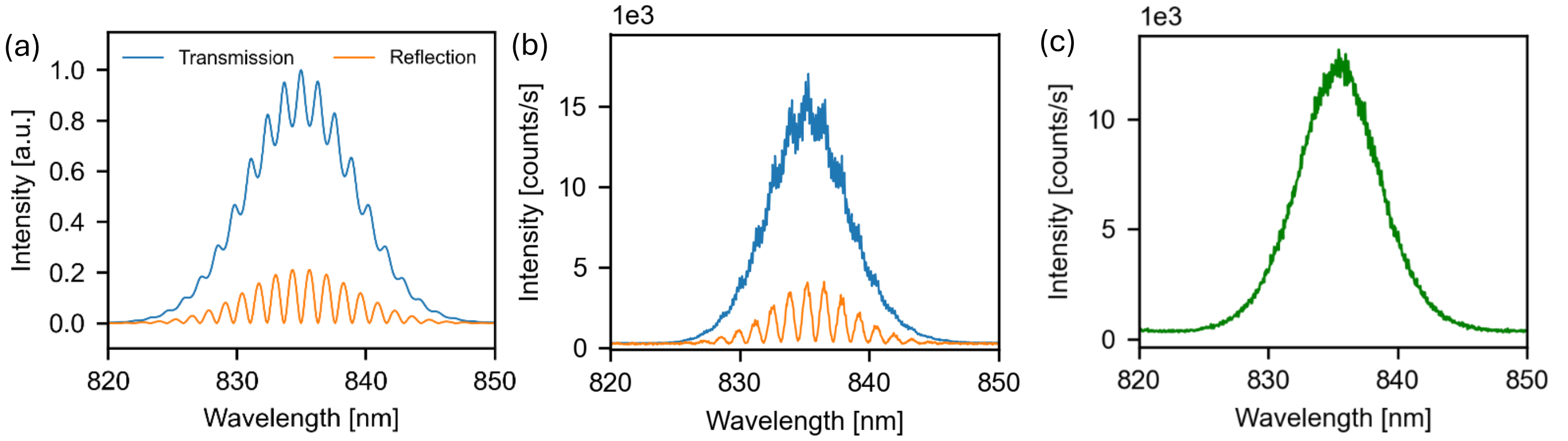

Figure R1: **Fabry-Perot interference from the substrate.** (a) Frequency domain model of Fabry-Perot interference of the pulsed pump laser at 835 nm due to the 175 µm thick substrate. (b) Transmission and reflection intensity of pump through and off the substrate, respectively. (c) Pump reflection off an InSb slab.

Our model qualitatively agrees with the spectral intensity of the pump measured through the substrate in transmission and in reflection in Fig. R1(b). This interference in intensity is preserved in the nonlinear process and observed in the FWM at the detector, which can be seen in Fig. 4 of our article.

I think the misunderstanding from the reviewer comes from considering the interference on a pulse-by-pulse basis. Instead, the modal content in the frequency domain (frequency comb) defines pulse shape and repetition rate across the entire pulse train through its Fourier relationship.

Additionally, the equivalent free spectral range in the space domain is $\Delta\lambda_{FSR} = \frac{\lambda^2}{2nL}$ and extracting the peak positions for Fig R1(b) allows us to calculate a L at 835 nm of 176$\pm$13.0 µm, which is in agreement with substrate thickness of 175 µm quoted by the manufacturer. Alongside Fig. R1(c), which shows pump reflection off bulk InSb, it is clear the interference is appearing in the pump due to the substrate.

Reviewer #2:

2.1. *The paper entitled "Phase Matching Free Sensing with Undetected Light Using a Nonlinear Metasurface" by Millar et al, demonstrates a method of classical sensing with undetected light using stimulated four-wave mixing (FWM) and a gold plasmonic nonlinear metasurface. The metasurface advantage enables phase-matching-free sensing, transferring amplitude and phase information from near-infrared (NIR) light (1500 nm) to visible light (580 nm). A Michelson-style interferometer is used, achieving over 50% visibility despite inherent reflections. Experiments include sensing through opaque silicon and modulating single-mode transmission with a knife edge. The spectral resolution reveals dispersion effects, enabling ultrafast sensing applications. The metasurface's subwavelength thickness and resonant properties eliminate phase-matching constraints, offering potential for sensing in the mid-infrared (MIR) range and ultrafast spectroscopy.*

The work is of significant interest and importance to the field, with the advent of metasurfaces being more readily used in sensing applications and for developing FWM methodologies without bulk nonlinear crystals. The work has sound analysis and interpretations, and provides sufficient detail for reproduction and theoretical understanding.

We would like to thank the reviewer for their positive comments.

Some issues that I feel need rectifying are highlighted below:

2.2 *It seems a little distracting on Page 2 in the introduction section, that the 2nd paragraph finishes with the statement of classical sensing being advantageous versus quantum sensing, but then paragraph 3 goes on to discuss the achievement of quantum sensing, before chapter 4 then goes back onto the topic of classical sensing being uses here. It's somewhat dissonant, so I think rewording these paragraphs to make it especially clear that you are not looking at a quantum sensing mechanism is needed.*

We have edited the introductory paragraphs to improve clarity. Each paragraph now introduces the following topics:

- Paragraph 1: Introduction to nonlinear interferometry
- Paragraph 2: Quantum nonlinear interferometry (induced coherence without induced emission)
- Paragraph 3: Achievements and drawbacks of quantum nonlinear interferometry (why stimulated emission is necessary when using metasurface sources)
- Paragraph 4: Classical nonlinearity interferometry (advantages over quantum implementation and upconversion sensing)
- Paragraph 5: What is achieved in the presented work; classical sensing using a nonlinear metasurface source

2.3 *Equation 1 would benefit with an additional =..... of the terms with αEpEs added to the right of the σ.*

E_{f1-} Is now written in terms of α_- as the reviewer has suggested. Eq. (1) reads:

$$E_{f1-} = \sigma_- = \alpha_- E_{p1}^2 E_{s1}^* e^{ik_f l}$$

2.4 *Could the authors add in the corresponding wavelengths as insets in to the subfigures for figure 2, this makes it easier to understand the visibility percentages when mentioning the different wavelengths of the pump and FWM wavelengths.*

Fig. 2 shows visibility and phase maps from FWM/signal interference before and after adding a silicon window to idler arm of the interferometer. The camera does not spectrally resolve the FWM emission and therefore the visibility and phase maps are taken across the entire spectrum centred at 580 nm. To clarify this point, the figure caption title has been updated to read:

"Visibility and phase of the FWM (signal) interference, centred at 580 nm, with and without a 240 μm silicon window in the seed (idler) arm of the interferometer."

2.5 *Line 198 states "Interestingly, a Fourier transform of the interferometer visibility and phase would reveal ultrafast transients of the object transmission with a resolution less than the pulse duration." The language here implies that it "would" reveal features but hasn't been done or shown in the manuscript. Could the authors comment on this, and if so include a FT plot highlighting these features?*

The reviewer is correct to ask for clarification on this point. The proposed experiment involves a pump-probe system within the seed (idler) arm of the interferometer, where the seed beam acts as the probe and a second pump beam is needed to excite a sample at the object plane. The nonlinear interferometer then allows the ultrafast dynamics uncovered by the probe to be transferred to the visible for detection on a silicon sensor.

Fig.R2 shows a diagram of such an experiment where the pump beam is split, with one path reaching the metasurface source, as before, and the other traversing a delay line before exciting the sample plane. The seed beam takes the same path as before. The nonlinear interference is observed at the camera and ultrafast transients of the material are excited by the pump light (835 nm), measured at the seed/probe wavelength (1500 nm) and detected in the visible (580 nm).

Delay line 1 (DL1) temporally aligns the seed to the pump at the metasurface and DL2 aligns the pump to the seed (probe) at the object plane. In a conventional pump-probe system the temporal resolution is limited by the probe pulse, but for the proposed interferometric pump-probe system the Fourier transform of the visibility and phase gives intra-pulse information with a spectral resolution limited by the wavelength of the probe.

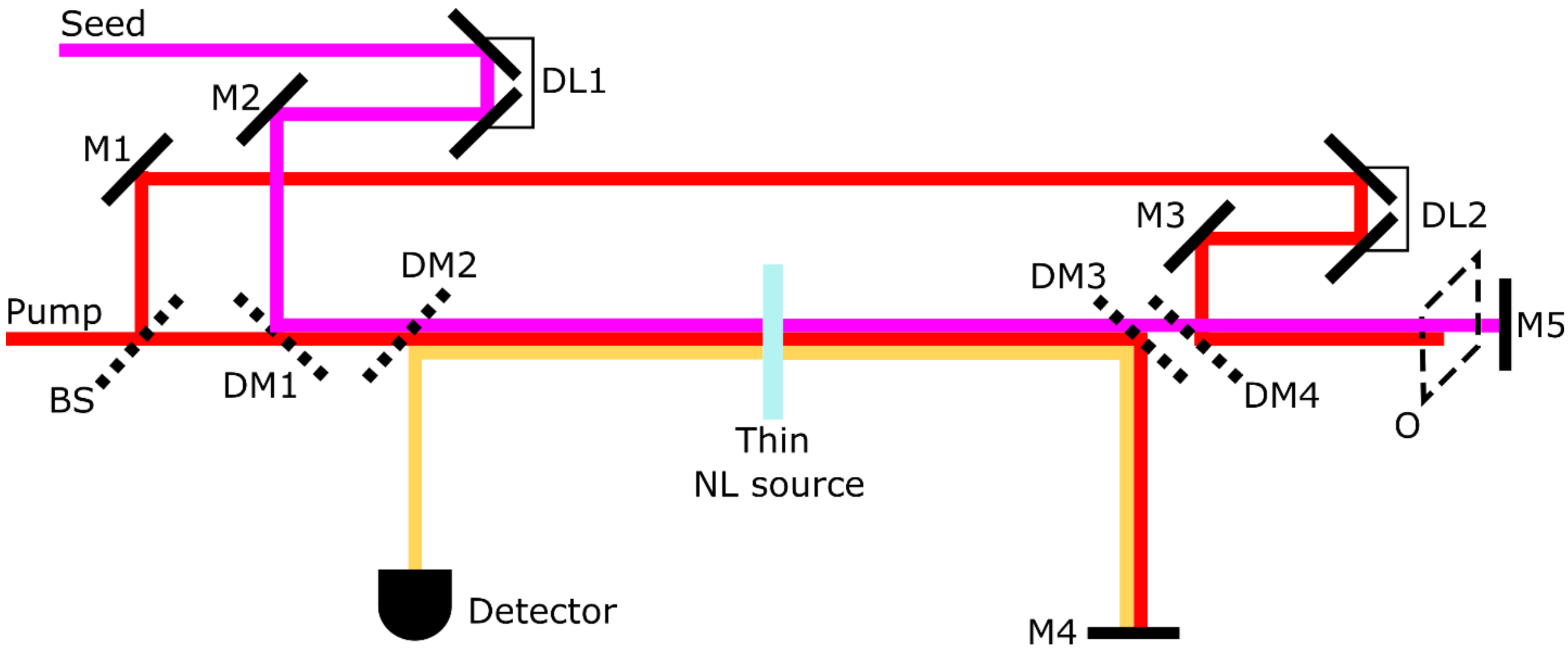

Figure R2: **The nonlinear interferometer system adapted for pump-probe measurements to uncover ultrafast transients of material at the object plane**. The pump beam is split, with one path used to generate FWM at the metasurface and the other used to excite material at the object plan, O. The information on the ultrafast dynamics of the material is transferred to the visible and observed in the interference at the detector. (DL – delay line, DM – Dichroic mirror, BS – beam splitter, M – mirror).

This is a more developed experiment than presented in the submitted article, which we reserve for the next stage of our research. Instead, the purpose of the statement quoted by the reviewer is to highlight a potential advantage of pulsed illumination. A Fourier transform of the interferograms in Fig.4(b) and (c) would not reveal ultrafast transients without the pump beam illuminating the object plane. To clarify this point, lines 219-221 have been rewritten:

“Interestingly, with the addition of a second pulsed beam to illuminate the object plane, a Fourier transform of the interferometer visibility and phase would reveal ultrafast transients of the object transmission with a resolution less than the pulse duration.”

Reviewer #3

3.1 *The authors developed a nonlinear interferometer utilizing a metasurface as nonlinear media and demonstrated sensing with undetected photons by exploiting stimulated four-wave mixing within the metasurface. Using the system, they measured the interference at the interferometer output and evaluated the visibility and phase. They also discussed the effect of bidirectional FWM emission and the existence of the Si sample on the visibility. While a paper already exists that uses a metasurface in the sample arm of the interferometer [Paterova et al. Nanophotonics, 10 1775(2021), applying a metasurface as nonlinear media for the nonlinear interferometer is novel and conceptually interesting.*

We would like the thank the reviewer for their work in reviewing our manuscript and for pointing out the novelty and conceptually interesting nature of this work.

3.2 *However, this paper insufficiently shows how a metasurface can benefit undetected photons techniques and improve their performance. The results do not show or discuss how the unique characteristics of a metasurface, bidirectional emission or phase-matching free features, which are not present in conventional nonlinear media, are beneficial for sensing with undetected photons. The data presented as proof of principle is also insufficient to demonstrate the potential of this method as a future imaging or spectroscopy technique. Therefore, unfortunately, I cannot recommend this manuscript for publication in Nature Communications.*

We thank the reviewer for their comment, but do not agree with the points made. Research can be impactful without showing immediate improvements over the current state of the art, especially when the novelty of the research lies in altering a fundamental element of a conventional system, in this case the nonlinear source of a nonlinear interferometer. Metasurfaces are of interest because they promise properties that extend past those of conventional materials. Therefore, demonstrating the distinct operation of nonlinear interferometry with a metasurface source, as a proof of concept, opens-up research avenues for applying these said properties to improve on conventional systems. This is undoubtedly valuable progress for the research field. Moreover, our work extends beyond metasurfaces to any thin nonlinear material where phase matching no longer applies. In our view, this approach opens this area of nonlinear interferometry to new investigations.

We respectfully disagree with the reviewer's claim that the presented work does not demonstrate the potential benefit of using a metasurface source for undetected photon techniques. As the submitted article clearly demonstrates that a custom made metasurface can be used as a nonlinear source within an interferometer, the research necessary to extend the spectral range beyond what is achievable with conventional nonlinear interferometry distils to metasurface development. This point is discussed in lines 245-248:

"In combination, pulsed illumination and stimulated nonlinear emission reduce the requirement for millimetre-scale exotic crystals and phase matching. The nonlinear interferometer system introduced here has the potential to reach any wavelength range where a laser and metasurface resonances are spectrally overlapped."

The phase-matching-free nature of the metasurface used in this submission was demonstrated by Yang et al. (DOI: 10.1021/acsphotonics.5c00764), through the tuneable spectral emission from the metasurface, and it felt unnecessary to re-demonstrate this beneficial characteristic. Bidirectionality is a by-product of phase-matching-free emission. However, as the reviewer has helped us understand, there are interferometer configurations where non-interfering fields can be removed. These interferometer schemes and accompanying theory have been added to the supporting information and are discussed in answer to point 3.6.

In Fig. 4 the technique is applied to spectroscopy, demonstrating its potential for such a measurement. Additionally, imaging is sensing across multiple modes and therefore a multimode seed beam would promote imaging capability. In response to point 3.3, we discuss how our system could be used for imaging and highlight that this does not change the distinct operation of the nonlinear interferometer with a thin-film metasurface source, which is a key message of our article.

Furthermore, with the addition of a second pump beam to excite the material at the mirror/object plane, the femtosecond pulsed illumination would facilitate intra-pulse ultra-fast pulse sensing and shaping with undetected light. While previous literature has demonstrated nonlinear interferometry with pulsed illumination,

a thin-film source will best preserve the transform limited pulse for operation on shorter time scales, as is discussed in answer to point 3.5.

The detailed points are as follows:

3.3 *Metasurfaces can exhibit resonance over a wide wavelength or wavevector range due to their phase-matching-free feature, which benefits imaging or spectroscopy applications. However, as the authors mention, the seed pulse restricts the FWM signal to a single mode, offsetting the advantages.*

The seed beam does restrict the modal content of the nonlinear emission, in comparison to spontaneous emission. However, this means the metasurface is tuneable across the broad wavelength and wavevector range of this spontaneous emission. Additionally, the spatial modes of the FWM (signal) could be increased with multimode seeding, which could be achieved with a diffuser in the seed path prior to the first pass through the metasurface. The application to spectroscopy was demonstrated in Fig. 4 and the spectral range of such a measurement could be extended by using a broader-bandwidth seed beam. It is important to note that these proposed changes to the seeding do not alter the operation of the thin-film nonlinear interferometer introduced in the submitted article. Furthermore, the metasurface itself can be tailormade for a specific spectral range, resonantly controlling where the nonlinearity occurs and giving a higher degree of control than that offered by nonlinear crystals. This has been shown in our previous work.

To highlight the tuneability of the system across the wavelength and wavevector range of the metasurfaces spontaneous emission, a sentence has been added to lines 237-239:

"Similarly, for spectroscopy, the system can utilise the broad spectral range of the resonant metasurface, either by tuning the seeding wavelength or illuminating with a broadband seed beam."

3.4 *I understand that a femtosecond seed pulse improves the brightness of the FWM signal and allows sensing with undetected photons in a realistic detection photon number and measurement time. In this case, this method needs to be compared to conventional femtosecond upconversion sensing [e.g., Ashik A. S. et al. Photonics Research 7 783 (2019)., Johnson and Diddams, Appl. Phys. B 107 31(2012)], in my opinion. What is the merit of using this seeded FWM interferometer against them? If a femtosecond light source is eventually required, one of the advantages of undetected photons methods, which is simple with a monochromatic pump source and a nonlinear medium, will be lost, and it will be no different from upconversion techniques. Additionally, the true value of undetected photon techniques is demonstrated in the mid-infrared or ultraviolet region; however, this method requires mid-infrared femtosecond seed pulses, which is a similar situation to that of upconversion techniques.*

The reviewer is correct to recognise that seeded nonlinear interferometry has similarities in implementation to an upconversion sensing system, the primary similarity being that a seed beam is required. Aside from this, a seeded interferometry system has all the advantages of an interferometric system over direct upconversion, regardless of CW or pulsed operation:

- Phase sensitivity
- Greater robustness to noise compared to a direct measurement of intensity
- Improved sensitivity at low transmission in the idler arm due to the square root of transmission relationship
- Immunity to thermal background noise and other incoherent background due to its coherent nature

Compared to quantum sensing with undetected photons, seeded nonlinear interferometry requires an additional light source at the sensing wavelength. However, the greater flux of photons comes with advantages over the quantum case:

- Improved signal-to-noise ratio as the modulation depth of interference is proportional to the number of seeding photons
- Greater coherence of the bi-photon state (CW)

The application of femtosecond pulsed illumination also presents advantages over quantum nonlinear interferometry and also CW seeded operation:

- Reduced dependence on highly nonlinear materials
- Broadened spectral range to wavelengths where resonant metasurfaces can be engineered and seed lasers are available

It should be noted that pulsed illumination limits the coherence time to the pulse duration, highlighting that choice of classical or quantum interferometry, or, CW or pulsed seeding, is one of practicality. For some applications it may be more practical to use an unseeded crystal based interferometer, but if there is no appropriate nonlinear material available, fast pulsed and seeded thin-film interferometers are an alternative solution.

From these comparisons, it is appropriate to say that seeded nonlinear interferometry is a distinct technique from both upconversion sensing and quantum sensing with undetected light, offering advantages over both. Some advantages over quantum nonlinear interferometry are stated in lines 46-48:

"The advantages of the classical variant over the quantum mechanical are an improved signal-to-noise ratio and greater coherence of the bi-photon state."

In the lines 48-51 the advantages of classical nonlinear interferometry over upconversion sensing are now included:

"Upconversion sensing also relies on stimulated nonlinear emission;[30,31] however, classical nonlinear interferometry distinguishes itself with phase sensitivity, stronger robustness to noise, enhanced sensitivity at low amplitude, and immunity to incoherent backgrounds."

In the conclusion, on lines 245-248, additional advantages of femtosecond pulsed classical nonlinear interferometry over quantum nonlinear interferometry are stated:

"In combination, pulsed illumination and stimulated nonlinear emission reduce the requirement for millimetre-scale exotic crystals and phase matching. The nonlinear interferometer system introduced here has the potential to reach any wavelength range where a laser and metasurface resonances are spectrally overlapped."

3.5 *The authors mention the potential for ultrafast measurements using the femtosecond seed pulses, but this is not a unique characteristic of this method. Some researchers have already demonstrated sensing with undetected photons using pulsed pump, signal, and idler [Kaur et al. Phys. Rev. Applied 22, 044015 (2024), Hashimoto et al. Commun. Phys. 7, 217 (2024)]. The pulse durations are picoseconds, but the hundreds of femtoseconds can be achieved by reducing the crystal length.*

Both papers referenced by the reviewer demonstrate FTIR spectroscopy within a nonlinear interferometer using pulsed illumination; Kaur et al. (2024) using a 5 ps laser and Hashimoto et al. (2024) using a 15 ps laser, both at 532 nm. With the integration of a beam illuminating the measured samples, both experiments could also probe ultrafast dynamics with a resolution set by the probe wavelength (see response 2.5), but do not demonstrate this. The reviewer correctly points out that a shorter crystal will lead to a shorter pulse duration of the signal and idler modes for the referenced systems. For our system, the signal mode is set by the seed beam, but a shorter source will better preserve the transform limited pulse, i.e. minimise walk-off and maximise the phase matching bandwidth. Therefore, both the spontaneous/high-gain systems and the seeded system are optimised for minimising pulse duration at the thin-film limit, highlighting the advantage of a nonlinear metasurface source over the bulk crystals used in the two references provided. This is a valuable advantage that should be mentioned in our article. Lines 221-224 read:

"With this in mind, the pulsed modes are expected to preserve their transform limited nature in our system; experiencing no temporal broadening from group velocity mismatch or phase matching bandwidth narrowing associated with bulk nonlinear material."

We would also like to point out the contemporary nature of the articles that apply pulsed illumination to nonlinear interferometry, which states advantages such as operating in the high gain regime [Hashimoto et al. (2024)], achieving greater sensitivity with increased photon numbers [Hashimoto et al. Adv. Quantum Technol. 8, 2300299 (2025)] and, as stated in the submitted article, facilitating ultrafast measurements [Kaur et al. (2024)]. As the latter article shares our perspective, this has been added as a reference to line 224.

3.6 *The bidirectional FWM emission is a unique and interesting feature of using a metasurface. The authors demonstrate that directivity results in reduced visibility compared to the ideal case when utilizing a conventional nonlinear medium, which is a disadvantage of using a metasurface. Are there any significant advantages of the bidirectional emission technique over the undetected photons techniques, such as simultaneous sample and background measurement? I suppose there would be a unique advantage to using a metasurface over conventional crystal-based interferometers, and I believe the advantage should be highlighted.*

The bidirectional emission is indeed an interesting feature of nonlinear interferometry with any thin-film source – either a metasurface or nonlinear film. In the presented work one of the four nonlinear fields does not interfere and only contributes to the background. In the supplementary information we have presented two additional systems. The first is a Mach-Zehnder interferometer with thin-film metasurface sources that is capable of reaching similar levels of visibility to conventional nonlinear interferometers by directionally filtering the nonlinear fields. The second is a Michelson interferometer where the thin-film source has been tilted to angularly misalign the nonlinear fields. This separates the interfering and non-interfering fields to improve visibility while also facilitating a background measurement of the nonlinear field.

Fig. R3 shows a schematic of a Mach-Zehnder interferometer. In the proposed setup, a pump and seed pass through two identical thin-film sources generating bidirectional emission at each. E_{p1} and E_{s1} are the pump and seed fields at the first source, which generate FWM mixing fields E_{f1+}in the forward direction and E_{f1-} in the backwards direction. E_{p2} and E_{s2} are the pump and seed fields at the second source that generate FWM

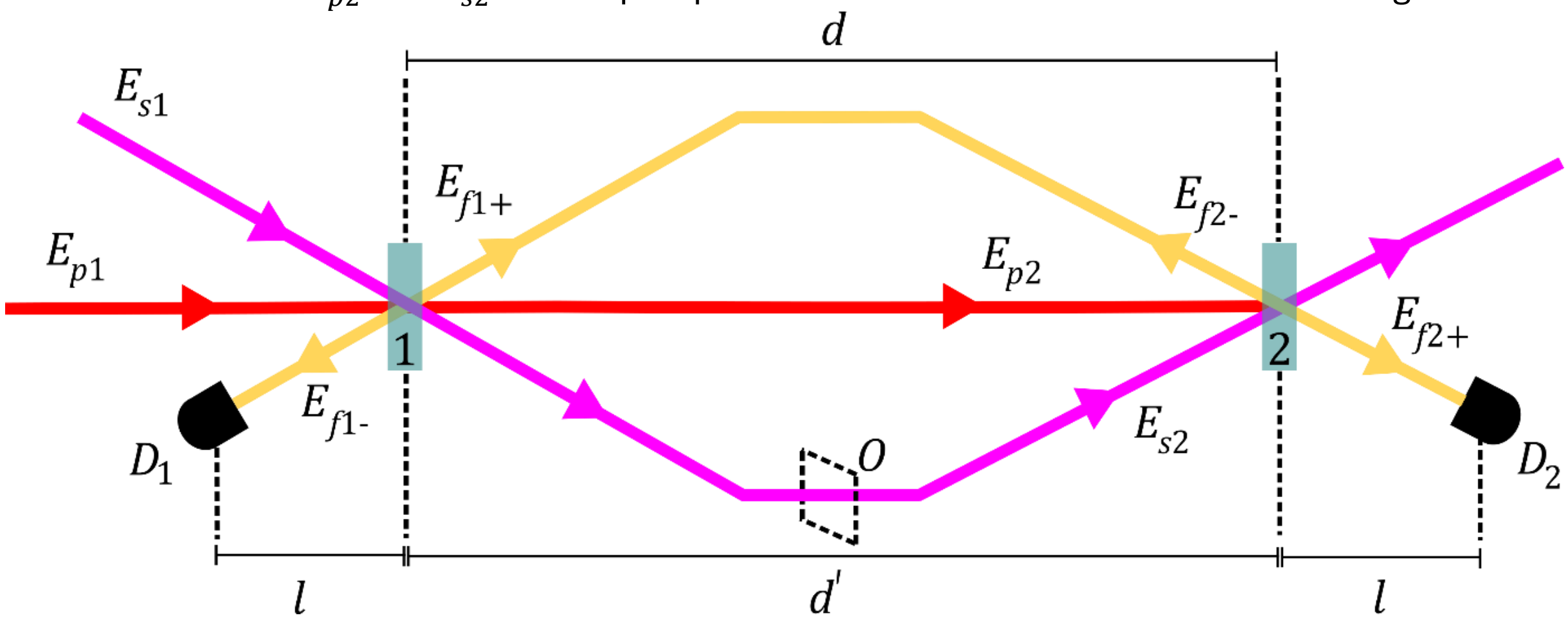

Figure R3: **A Mach-Zehnder interferometer with two identical thin-film nonlinear sources**. The fields $E_{f1\pm,f2\pm}$ represent the bidirectional emission at the first and second source, respectively. Detector 1, D_1, measures the backwards emitted FWM fields and detector 2, D_2, measures the forward emitted fields. Inside the interferometer, the FWM and pump travel path d and the seed (idler) travels path d'.

fields E_{f2+}in the forward direction and E_{f2-} in the backward direction. Detector 1, D_1, measures the backward-generated fields and Detector 2, D_2, measures the forward-generated fields.

The full derivation in SIII of the supporting information of the revise manuscript reaches definitions of the four fields generated per pulse within the interferometer.

$$E_{f1-} = \sigma_-$$

$$E_{f1+} = \sigma_+ t_f e^{ik_f d}$$

$$E_{f2-} = \sigma_- t_f t_s t_p^2 t e^{i\gamma} e^{i\psi} e^{i2k_f d}$$

$$E_{f2+} = \sigma_+ t_s t_p^2 t e^{i\gamma} e^{i\psi} e^{ik_f d}.$$

As before we have temporal/phase windows defined by the paths taken by the various fields. The two forward generated fields are overlapped at $k_f d$, while the two backwards generated fields are separated from one another by $2k_f d$. However, as before, the E_{f1+} field reflects off source 2 towards D_1 and is overlapped with the E_{f2-} field. This means at D_1 we have the intensity

$$D_1 = \left|E_{f1-}\right|^2 + \left|E_{f2-} + r_f E_{f1+} e^{ik_f d}\right|^2 = |\sigma_-|^2 + \left|\sigma_- t_f t_s t_p^2 t e^{i\gamma} e^{i\psi} e^{i2k_f d} + t_f r_f \sigma_+ e^{i2k_f d}\right|^2.$$

Which leads to a normalised intensity of

$$N_1 = \frac{D_1}{\sigma_+^2} = \eta^2\left(1 + T_f T \kappa\right) + T_f R_f + 2\eta T_f \sqrt{\kappa R_f T}\cos(\psi + \gamma).$$

Here, $T = |t|^2$ and $R = |r|^2$, $\kappa = T_P^2 T_s$ and $\eta = \sigma_-/\sigma_+$. This shows we expect to see interference at D_1, rather than temporally separated nonlinear fields. The visibility of this interference can be calculated using $V = \frac{N_{max} - N_{min}}{N_{max} + N_{min}}$.

$$V_1 = \frac{2\eta T_f \sqrt{\kappa R_f T}}{\eta^2\left(1 + \kappa T_f T\right) + T_f R_f}$$

At D_2 we have interference between the forward emitted fields.

$$D_2 = \left|E_{f1+} + E_{f2+}\right|^2 = \left|\sigma_+ t_f e^{ik_f d} + \sigma_+ t_s t_p^2 t e^{i\gamma} e^{i\psi} e^{ik_f d}\right|^2$$

The normalised intensity of these fields is

$$N_2 = \frac{D_2}{\sigma_+^2} = T_f + \kappa T + 2\sqrt{\kappa T T_f}\cos(\gamma + \psi)$$

and the visibility from this normalised intensity is

$$V = \frac{2\sqrt{\kappa T T_f}}{T_f + \kappa T}.$$

We have arrived at a scenario where D_1 measures interference with a similar visibility limit as the Michelson configuration in article's main text, but D_2 measures interference capable of reaching 100% visibility, similar to conventional nonlinear interferometers. Because E_{s2} travels through the object plane to generate E_{f2+}, the interference in the backwards direction contains transmission information on the object at O. It is worth noting the interfering fields that reach D_1 will also reflect off source 1 and reach D_2, but assuming coherence between the fields, this should not impact the maximum achievable visibility.

Fig.R4 shows a tilted metasurface, assumed to be inside a Michelson interferometer for this derivation, with the generated and reflected nonlinear fields labelled. The metasurface is tilted at an arbitrary angle θ from normal incidence with the optical axis, shared by the collinear pump and seed (idler) beams. At the first pass, the E_{f1+} field is emitted along the optical axis, but the E_{f1-} field is emitted $-\theta$ from the sample normal (2θ from the pump-seed optical axis). A similar scenario is expected at the second pass, where E_{f2-} is emitted along the

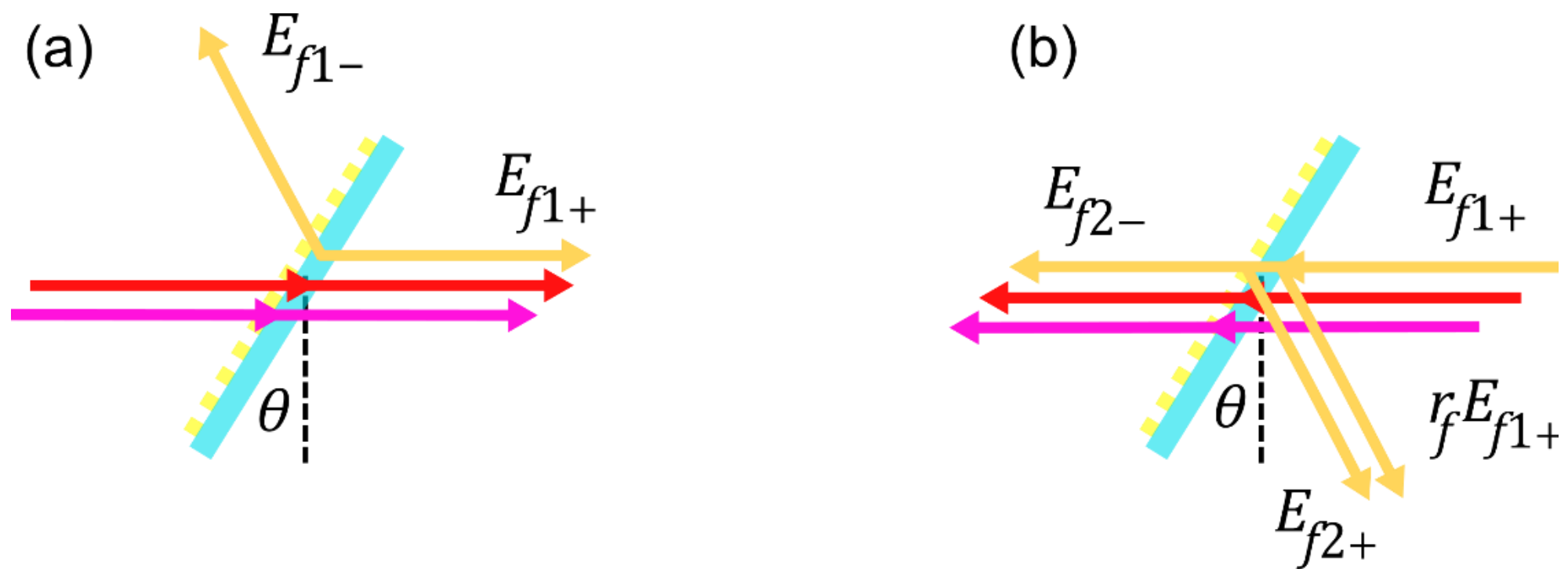

Figure R4: **Angular misalignment of FWM (signal) fields when the metasurface source is tilted inside a Michelson interferometer**. (a) FWM (signal) fields at the first pass, where the E_{f1-} field is reflected away from the optical axis of the colinear pump and seed. (b) FWM (signal) fields at the second pass, where the secondary interference pattern is reflected away from the optical axis.

optical axis, but the E_{f2+} field is emitted at $-\theta$. The first pass field is reflected off the metasurface ($r_f\, E_{f1+}$) at the same angle. Therefore, the tilted metasurface introduces three paths, one for each of the three temporal/phase windows previously identified. As each set of fields is now separated angularly rather than by their phase, we define the nonlinear amplitude and FWM (signal) fields without the unnecessary path information.

$$\alpha_\pm E_p^2 E_s^* e^{i\left(2\phi_p(\omega)-\phi_s(\omega)\right)} = \tilde{\sigma}_\pm e^{i\phi_f(\omega)} = \sigma_\pm$$

$$E_{f1-} = \sigma_-$$

$$E_{f1+} = \sigma_+$$

$$E_{f2-} = t t_s t_p^2 \sigma_- e^{i\gamma} e^{i\psi}$$

$$E_{f2+} = t t_s t_p^2 \sigma_+ e^{i\gamma} e^{i\psi}$$

By terminating each path with detectors, the intensity of each set of fields can be measured separately.

$$D_1 = \left|E_{f1-}\right|^2 = \sigma_-^2$$

$$D_2 = \left|t_f E_{f1+} + E_{f2-}\right|^2 = \left|\sigma_+ t_f + t t_s t_p^2 \sigma_- e^{i\gamma} e^{i\psi}\right|$$

$$N_2 = \frac{D_2}{\sigma_+^2} = T_f + T T_s T_p^2 \eta^2 + 2\eta\sqrt{T_f T_s T_p^2 T}\cos(\gamma+\psi)$$

$$D_3 = \left|E_{f2+} + r_f E_{f1+}\right|^2 = \left|t t_s t_p^2 \sigma_+ e^{i\gamma} e^{i\psi} + r_f \sigma_+\right|$$

$$N_3 = \frac{D_3}{\sigma_+^2} = R_f + T T_s T_p^2 + 2\sqrt{R_f T_s T_p^2 T}\cos(\gamma+\psi)$$

Again, $T = |t|^2$ and $R = |r|^2$ and $\eta = \sigma_-/\sigma_+$. D_1 clearly measures a single field with no interference. Two fields are expected at D_2 and D_3, and the normalised intensity at both detectors contain direct current and oscillating terms. A theoretical visibility can be define using the same equation from above, $V = \frac{N_{max}-N_{min}}{N_{max}+N_{min}}$.

$$V_2 = \frac{2\eta\sqrt{\kappa T_f T}}{T_f + \kappa\eta^2 T}$$

$$V_3 = \frac{2\sqrt{\kappa R_f T}}{R_f + \kappa T}$$

Both V_2 and V_3 can reach 100% visibility, although may be limited by the optical properties of the applied metasurface. V_3 is derived from interference between two fields emitted in the positive direction and is therefore independent of η. In conclusion, using a tilted metasurface source could result in visibility that rivals conventional nonlinear interferometry at D_2 and D_3, while simultaneously monitoring the nonlinear emission that does not reach the object plane at D_1. This is a unique advantage of using a metasurface or thin-film source over conventional bulk crystals.

The proposed system has been included in the supporting information and discussed in the main text on lines 141-146:

"We note that the reduction in the maximum visibility when using a metasurface source is predominantly due to the E_{f1-} field. The FWM (signal) fields can be directionally filtered in a Mach-Zehnder interferometer to achieve a maximum visibility that is theoretically equivalent to conventional nonlinear interferometers. Alternatively, tilting the metasurface to misalign nonlinear reflections also maximises visibility while simultaneously allowing the nonlinear emission to be monitored. These configurations are discussed theoretically in Sections SIII and SIV of the SI."

3.7 *Their developed system, consisting of two curved mirrors with a Si sample in the seed arm, insufficiently shows the potential for future spectroscopy or imaging applications. The visibility drop shown in Figure 2 is not mainly associated with the Si's transmittance but rather with the change in beam divergence after adjusting the temporal delay, in my understanding. The phase change is also not directly related to the absolute Si phase. Although this method operates in a single-mode regime, it may be necessary to collimate the seed beam with a lens (and focus) and irradiate it onto a sample, assuming imaging/spectroscopy applications.*

The measurement through the Si window is valuable because it shows interference through the same material that is then used to detect it, highlighting the undetected nature of the technique. It is not an attempt to measure the phase shift introduced by the Si window nor the drop in transmission, as the transmission-visibility curve is investigated in Fig. 3(f).

It was made clear on lines 163 and 164 that the reduction in the maximum visibility with the introduction of the silicon window is predominantly due to divergence of the seed necessary to achieve a temporal overlap with the pump at the second pass:

"Re-establishing temporal overlap is likely to have reduced nonlinear emission on the second pass, and in turn, reduced visibility."

Similarly, we stated on lines 162-163 and 165-166 that the phase shift is not a measurement of the Si window due to the optical path length change being greater than the coherence length of the interference:

"The path length introduced by the Si window is ~1.2 mm; greater than the coherence length of interference."

"A further consequence of this realignment is that a direct measure of the phase change cannot be taken from Fig. 2(c) and (d)"

This is expected as the coherence length of interference inside a pulsed laser interferometer is limited by the pulse duration.

The changes to the optical system suggested by the reviewer will decouple the focal position from the temporal alignment, removing the dependence of visibility on optical path length, but will not improve the coherence length of interference. As the dependence of visibility on seed (idler) transmission at the object plane for sensing and imaging applications is demonstrated in Fig. 3(f), we do not agree that the proposed experiment offers a substantial improvement on the submitted work. Furthermore, it is not a novel design, having essentially already been implemented in the articles highlighted by the reviewer: [Kaur et al. Phys. Rev. Applied 22, 044015 (2024), Hashimoto et al. Commun. Phys. 7, 217 (2024)].

【Minor points】

3.8.1 *I suggest that the authors describe the source and detector parameters, including the pump and seed powers (or pulse energy), the generated FWM signal power, the detected signal power in the camera, and the exposure time/ measurement time of the detector. The information may help readers understand that the brightness and signal-to-noise ratio are improved by the seeding effect.*

The peak powers of the pump and probe have been included on lines 72-73:

"The peak powers delivered by the pump and seed fields are 14.5 kW and 80.0 W, respectively."

The resulting FWM power reaching the camera from a single path is now included on lines 79-83, along with the camera settings:

"From a single pass through the metasurface, the peak power of FWM (signal) emission is 4.81 nW across a 1.17 mm diameter spot at the camera. The photon conversion efficiency relative to the seed beam is estimated to be 1.18×10^{-11}. When the seed beam is blocked, no FWM (signal) is observed at the camera using typical settings of 800 ms exposure time and 600× EM gain."

3.8.2 *I guess that the authors describe the equations assuming that the spontaneous emission caused only by the pump is small enough and the related quantum interference is negligible. If it is so, I recommend that the authors mention it.*

Yes, this is correct and should be stated. Lines 98-99 now read:

"The spontaneous emission is assumed to be negligible in comparison to the stimulated."

3.8.3 *I am unsure of "homodyne advantage" in this context, written in Line 56. Do the authors mean the nonlinearity of the visibility vs. transmissivity due to the existence of the seed pulse?*

The homodyne advantage refers to the sensitivity to seed field amplitude leading to square root dependence of visibility on seed intensity and object transmission within the interferometer. This is not unique to a seeded system, as this nonlinear relationship also appears in nonlinear interferometry with spontaneous emission.

3.8.4 *I am unsure of "signal interference independent of pump-seed delay" in Line 57, and it is not discussed in detail in the latter part. The interferometric visibility depends on the coherence time of the pump. If the authors meant that the interference range can only be determined by the idler optical path-length difference in the time-domain case, it has already been discussed in the previous work [Kaur et al. Phys. Rev. Applied 22, 044015 (2024)].*

The term pump-seed delay refers to the temporal alignment between the pump and seed before the interferometer, which is controlled by the delay line plus piezo and stepper motor shown on the top right hand side of Fig.1(a). The statement quoted by the reviewer therefore means that the temporal overlap of the pump and seed outside the interferometer does not affect the interference inside the interferometer within the temporal range that leads to observable FWM emission at the metasurface (naturally, if the pump and seed are delayed too much, there would be no FWM). The key point is that the relative delay of pump and seed, do not affect the internal phase measured by the nonlinear interferometer. This is important, as it allows the laser chirp to be exploited to collect the spectrograms in Fig. 4. This is not the same point as discussed by Kaur et al. as theirs is a quantum system without a seed beam.

To clarify our meaning here, line 62 has been included:

"...signal interference independent of pump-seed delay outside the interferometer..."

In lines 200-204, the pump-seed delay is discussed in greater detail:

"The piezo-controlled seed delay line, positioned before the 1 μm short-pass dichroic, is scanned across a 145 μm (483 fs) range with 2.5 μm (8.3 fs) steps, centred at 0 μm where the pump and seed are at their best temporal overlap that maximises the FWM (signal) emission in Fig. 4(a). The FWM (signal) spectrum blue shifts as the delay position moves from negative to positive, indicating chirp in the pump and seed pulses."

3.8.5 *A signal pulse can be generated when the pump and seed beams are reflected off the metasurface, enter the interferometer again, and then hit the metasurface again. Is the signal weak enough to be ignored?*

Yes, this signal emission due to pump and seed reflections is weak enough to be ignored, due to the FWM (signal) amplitude being dependent on the square of the reflected pump amplitude. Using the measured optical properties of the metasurface in Table SI of the SI, we estimate the FWM amplitude generated by the reflected pump and seed to be a factor of $r_p^2 r_s \approx 0.157$ smaller than the corresponding FWM generated directly by the pump and seed at the metasurface. Here, absorption has been ignored making this factor an overestimate. In comparison, the two FWM fields with phase $4k_f d$, E_{f2+} and the linear reflection of E_{f1+} from the metasurface $r_f E_{f1+} e^{2ik_f d}$, have commensurate amplitudes due to transmission and reflection coefficients of 0.48 and 0.31, respectively. As only one additional field is observed at the camera, we attribute it to the linear reflection of the E_{f1+} field.

Linear reflections of the pump, seed and FWM continue to create temporal overlap at $mk_f d$, where m is an even integer. However, this estimate of first set of reflected fields shows there will be a mismatch in amplitudes and allows us to approximate the interfering fields to the first reflected FWM field.

This approximation has been made clear in lines 118-120 in the main text:

"Linear reflections of the pump and seed beams by the metasurface have been omitted due to nonlinear scaling of FWM emission."

On lines 61-64 the SI has also been edited:

"Linear reflections of the pump, seed and FWM will create successive temporal overlap at $mk_f d$ where m is an even integer. However, the nonlinear scaling of FWM emission with pump and probe amplitude allows the model to be approximated to the first FWM reflection."

3.8.6 *I recommend that the authors add scale bars to the images in Figures 1 (f-h) and 2.*

Scale bars have been added to Fig.1(f-h) and their 2 mm length noted in the figure caption. The horizontal axis of Fig.1(i) has been edited to show position along the cameras horizontal (x-axis) in millimetres, matching the lines marked in Fig.1(f-h).

3.8.7 *Why does the interferometric visibility in Figures 2(a) and (b), especially the high-visibility part in the center, have a non-uniform distribution?*

The nonuniform distribution is due to the two interference patterns that are separated in time but overlapped at the camera within its exposure time: 800 ms $\gg 2d/c$. These two interference patterns do not coherently sum at all points in k-space across the camera's field of view. Instead, there are regions of coherent summation and regions of incoherent summation. The latter leads to destructive regions where the interference patterns are exactly π out of phase. As the interference measurement is single mode, only the coherent regions with the highest visibility are necessary to consider.

3.8.8 *I suggest that the authors explain the details of the axes in Figure 3(e). I am unsure of where the knife-edge position is, the meaning of 0.00 mm in this context, and the meaning of X-axis. Is the X-axis image size on the camera?*

The knife edge is stated to be at the object plane in the caption of Fig. 3. The position 0.0 mm is set so that the knife-edge is not obscuring the idler beam. As the knife edge is shifted, it covers the idler beam with the relative displacements shown. The indicated x-axis is that of the camera. This shows the shape of the visibility of the scanned object along the knife edge scan axis. The caption for this figure currently reads:

"(e) Experimental average visibility measured at knife edge positions that reduce seed (idler) transmission. The knife edge is brought along the horizontal axis of the object plane and visibility is averaged across three rows of pixels"

To reduce the chances for misunderstanding, a section has been edited in lines 186-189 of the main text to read:

“By scanning a knife edge horizontally across the object plane of the interferometer we can measure a reduction in visibility across the horizontal (x-axis) of the camera, shown in Fig.3(e). The knife edge starts by not obstructing the seed (idler) beam at 0.00 mm to fully obstructing it by 6.25 mm, and visibility is calculated at each knife edge position.”

Also, the x-axis of Fig.3(e) is now labelled “Camera x-axis” to match Fig.1(h) and the tick labels mark the region of the camera axis that has been cropped to show just the interfering fields. The original scale on Fig. 3(e) had not accounted for the binning used and therefore had an incorrect range, the new range is correct.

SECOND ROUND REVIEWER COMMENTS

Reviewer #1 (Remarks to the Author):

In the rebuttal letter, Millard et al. respond to my main comment, concerning the usefulness of stimulated FWM in their plasmonic metasurface for sensing with undetected photons. While the potential of this work to be impactful for quantum sensing is arguable, I agree that the observation of interference in an SU(1,1) interferometer based on a metasurface is indeed a breakthrough. Therefore, I am, in principle, not objecting against the publication in Nature Communications.
But I still have doubts about certain technical points.

We thank reviewer #1 for their support of the manuscript's publication. The technique presented is the classical variant of sensing with undetected light. While the mechanism of the nonlinear optical process is different, the interferometric scheme remains nearly identical and understanding of thin-film sources within such an interferometer is a benefit to both quantum and classical implementations. For example, spontaneous four-wave mixing (Son et al. *Opt. Lett.* **2025** *50*, 4434-4437) and spontaneous parametric down conversion (Tomás Santiago-Cruz et al. *Science* **2022** *377*, 991-995) have both been observed from subwavelength sources. Such sources could eventually be integrated into a quantum sensing SU(1,1) interferometer, and the discoveries highlighted in our manuscript will undoubtedly be applicable.

1. To my previous comment about Fig.2, the authors insist that panels a,b show the visibility and not the interference pattern, as it looks like. But then, why does the visibility oscillate approximately the same way as the cos(phi) function? It is supposed to be the factor in front of the cosine, as described in the paragraph above the figure.

The answer to the reviewer's point highlights a significant distinction between thin-film nonlinear interferometry with a pulsed laser and conventional nonlinear interferometry. The fringes seen in the visibility in Fig. 2 are not due to the $\cos(\phi)$ function in Eq. (6), but due to the camera detecting two temporally separated (and spatially different) interference patterns. In Eq. (5) we identify two interference patterns, primary and secondary, that are time-averaged at the camera but that are not overlapped in time, with the secondary interference pattern appearing due to linear reflection from the metasurface. The camera therefore incoherently sums their intensities ($I_{sum} = I_{1^{st}} + I_{2^{nd}}$), rather than the coherent sum of electric field amplitudes which leads to interference (e.g. primary interference pattern $I_{1^{st}} = \left|E_{f1+}^2 + E_{f2-}^2\ \right|$). There is also a single field E_{f1-} which does not interfere. To reach the visibility equation, Eq. (7), we have assumed the two sets of interfering fields arrive in phase at the camera. In the experiment, there are regions of in-phase incoherent summation and regions of out-of-

phase incoherent summation across the camera's field of view. This creates an interference like pattern in the intensity measurement at the camera. To be clear, this is not optical interference because the two sets of interfering fields do not arrive at the camera at the same time; instead, it can be considered interference in time-averaged intensity at the camera.

Consider the following simplified example of this phenomenon. The primary (dashed line) and secondary (dotted line) set of interfering fields are modelled in one spatial dimension as cosine functions with equal amplitude in Fig. R1, showing the intensity of each interference and their incoherent sum (solid line) as would be measured at a detector. At zero relative phase shift, $\Delta\phi$, between the two sets of interfering fields the calculated visibility of the sum is identical to that of a single set of interfering fields, although the modulation depth (contrast) increases. At $\Delta\phi = \pi 2/3$, the sum has reduced visibility and at $\Delta\phi = \pi$ it appears to have zero visibility. For the latter case, both sets of interfering fields have 100% visibility but cancel one another out in the time-averaged intensity at the camera. In reference to Fig. 2 of the manuscript, across a given axis of the detector, $\Delta\phi$ (between the two distinct interference patterns) changes with the spatial frequency, cycling through maximum and minimum visibility in the intensity summation. In our current system we cannot separate the two interference patterns, however, this could be achieved be tilting the metasurface (Sec. SIV), building a Mach-Zehnder interferometer (Sec. SIII), or using a time-gated detector.

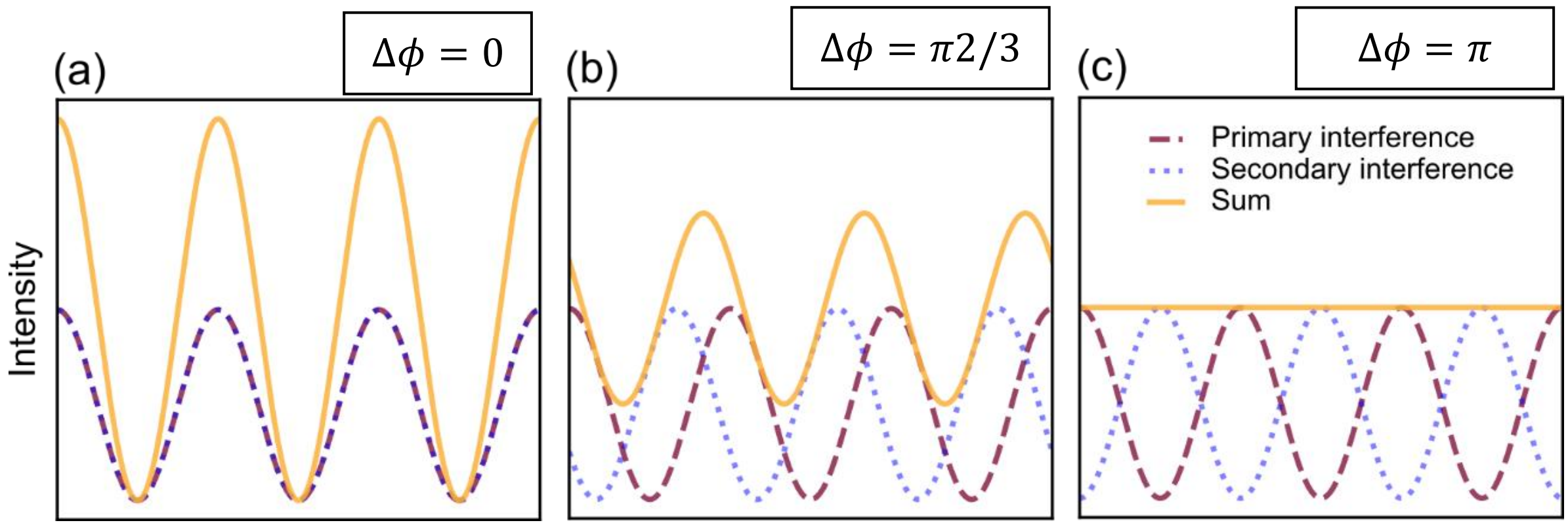

Fig. R1: **Incoherent time-averaged intensity of the two sets of interfering fields at the detector in one spatial dimension.** (a) Maximum visibility when the two sets of interfering fields are in phase. (b) Reduced visibility when partially out of phase. (c) Minimum visibility when π out of phase.

The regions of maximum visibility in Fig.2 represent the scenario modelled by Eqs. (6) and (7). To benefit understanding we now state on line 121:

"Assuming the pulses arrive in phase, the detected intensity normalised…"

And have added a paragraph across lines 155-164:

“In the theoretical description above, the two sets of temporally separated interfering pulses were assumed to have no in-plane phase variation across the camera. Experimentally, however, the two sets of interfering pulses create two distinct spatial interference patterns on the camera. Each pattern comes from intra-pulse electric field interference, but the time averaged camera image is formed from the incoherent sum of the two overlapping patterns from each pulse. This is the origin of the interference-like features observed in Fig. 2(a) and (b), as well as the phase dislocations in (c) and (d). It is important to emphasize that this is not optical field interference, as the two pulses of interfering fields only incoherently sum at the camera due to the exposure time being much greater than their temporal separation. As discussed in Sections SIII and SIV of the SI, such features could be removed by isolating a single set of interfering fields.”

2. I did not notice it before, but the abstract says: ‘...we report classical sensing with undetected light octave spanning stimulated four-wave mixing from a plasmonic metasurface.’ I do not see any octave-spanning spectrum here.

This is not referring to the bandwidth of the FWM (signal) emission but the spectral separation between the seed (idler) and FWM (signal). We realise the phrasing in the abstract is not quite correct and so we have changed it to:

“...we report octave-spanning classical sensing with undetected light using stimulated four-wave mixing from a plasmonic metasurface.”

As the sensing wavelength is 1500 nm and the detection wavelength is 580 nm, this spans almost two octaves and therefore can be described as octave spanning.

3. In the recent paper Son et al., Opt. Lett. 50, 4434 (2025), it is shown that FWM from a substrate can compete with the FWM from a strongly nonlinear film. Did the authors check that their substrate does not contribute to the effect?

The geometry of the constituent resonators of the metasurface are such that the incident pump and seed beams are cross polarised, with the seed polarisation aligned to the longitudinal axis of the bars and the pump polarised along the transverse axis to excite only the disc resonators. When beams illuminate the bare substrate, their orthogonal polarisation means they do not mix and no FWM emission is observed at the camera. Fig. R2(a) shows intensity at the camera for this exact scenario, with curved mirror 2 within the interferometer blocked and therefore collecting transmitted and reflected light from the first pass only. When the beams are co-polarised mixing does occur in the substrate and FWM is measured at the camera, Fig. R2(b). Although, as the substrate is 175 μm thick, the borosilicate would not be considered a thin film source.

Fig. R2(a) can be compared to the FWM emission from the metasurface shown in Fig. 1(h) of the manuscript, with some important clarifications. Fig. 1(h) was collected with 800 ms exposure time and 600x EM gain with a binning factor of 2, while Fig. R2(a) was collected with 1 s exposure time and 400x EM gain but with no binning applied. Despite these differences, the presence of the metasurface is seen to enhance the nonlinear emission. Furthermore, the previous studies of this metasurface measured the relative FWM emission from the substrate under cross polarised illumination (Gennaro et al. *ACS Photonics* **2018** *5* (8), 3166-3171) and demonstrated that the symmetry of the unit cell was a necessary consideration for functional nonlinear emission (Gennaro et al. *Nano Lett.* **2016** *16* (8), 5278-5285), and therefore that the substrate alone was not producing a significant signal.

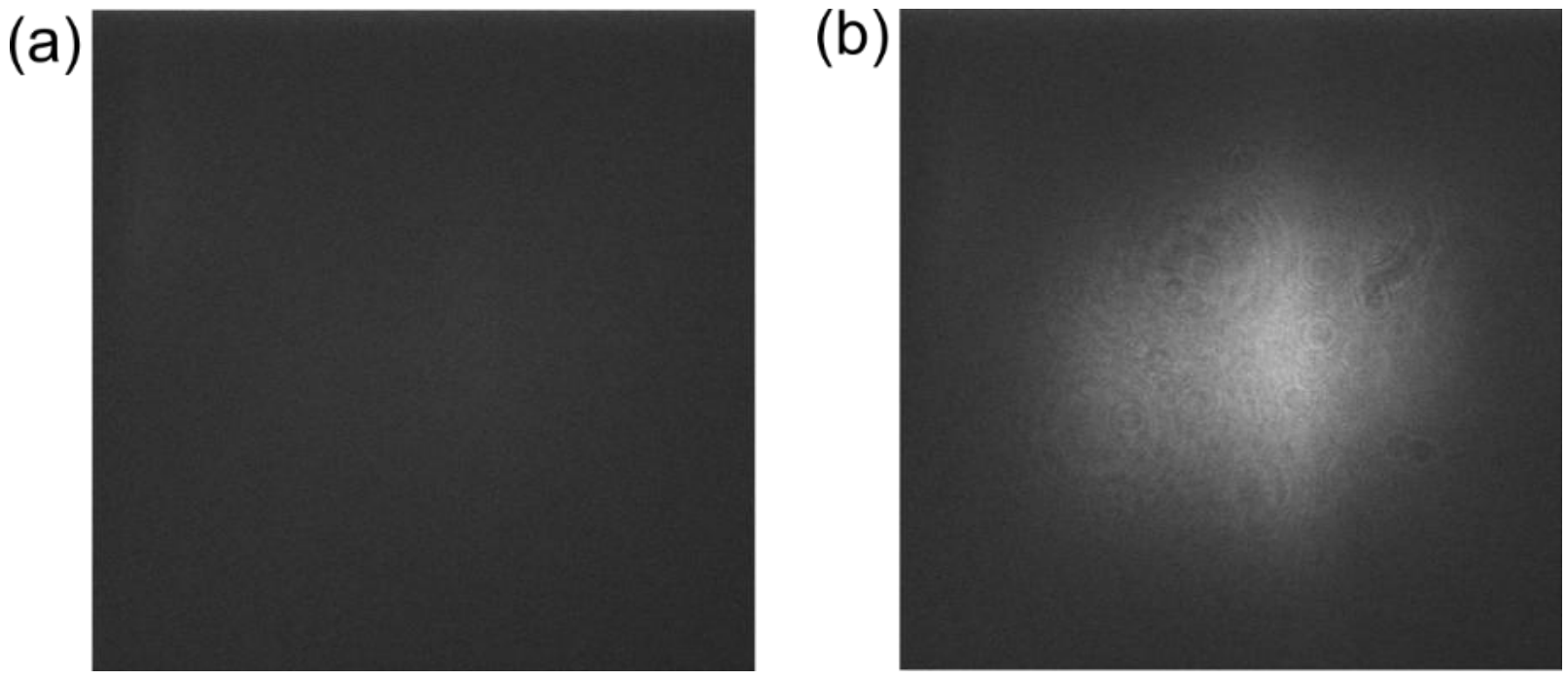

Fig. R2: **FWM emission from the SiO_2 substrate.** (a) Intensity at the camera when the pump and seed are cross-polarised and only the bare substrate is illuminated. (b) Intensity at the camera when the beams are co-polarised. Both images collected with 1s exposure time and 400x EM gain.

Reviewer #3 (Remarks to the Author):

I appreciate the authors' careful efforts to address all of my comments, both major and minor, and I believe that the revisions have significantly improved the manuscript. However, I remain unconvinced that one of my main concerns has been adequately addressed, namely, whether this proof-of-principle experiment demonstrates the capability for sensing with undetected photons.

The revised manuscript provides a thorough and well-written discussion of the characteristics of metasurface-based nonlinear interferometry, including its theoretical foundations. It explains that a Mach-Zehnder configuration can achieve near-100% visibility, and that a similar improvement can be obtained by tilting the metasurface. The tilted geometry further enables simultaneous detection of nonlinear emission, which is particularly interesting, as it may offer capabilities not available in conventional approaches. In addition, the authors emphasize the advantages of metasurfaces, including the ability to engineer the wavelength, bandwidth, and spatial mode of

biphotons without being constrained by phase-matching conditions, thereby further characterizing this platform. The ease of maintaining transform-limited pulses is also noteworthy and may indeed provide advantages over conventional methods. Overall, these discussions are highly informative and strengthen the manuscript.

We appreciate the reviewer's assessment of the modified manuscript and recognition of the work's strengths. Indeed, we are grateful for the reviewer's input, which we also believe has improved the manuscript. We hope to answer their remaining concerns in the following response.

Nevertheless, I still do not think that the presented proof-of-principle experiment convincingly demonstrates applicability to sensing with undetected photons. First, the results shown in Fig. 2 appear to primarily confirm the basic operation of the undetected-photons scheme, rather than demonstrating sensing. Although the authors state that "in Fig. 4 the technique is applied to spectroscopy" and that "the application to spectroscopy was demonstrated in Fig. 4," the experiment in Fig. 4 essentially extracts the fine spectral fringe arising from an etalon effect generated when the pump propagates through a borosilicate substrate. While frequency-resolved measurements are indeed performed, they do not constitute genuine spectroscopic sensing of a sample, such as the detection of idler absorption or phase shifts induced by a target object, nor are the results quantitatively evaluated or compared with known reference values. Although the spectrogram is useful for assessing dispersion within the system, it does not directly evaluate an object's sensing capability in the context of the undetected photons method.

Broadly, optical sensing is the measurement of a change in the amplitude and/or phase of light observed by a detector in the spatial or spectral domain. Regarding sensing with undetected light, the change must occur in the idler and be measured in the signal. According to this definition, Figs. 1, 2 and 3 demonstrate sensing, and due to the configuration of the interferometer and nonlinear source, demonstrate sensing with undetected light. While Fig. 4 does demonstrate application to spectroscopy, the reviewer is correct to point out that we do not spectrally measure a sample at the object plane and in that regard, we have not sensed in the spectral domain. However, as discussed in the main text, this was not the purpose of Fig. 4. In response to the reviewer's concerns we will discuss the purpose of each figure in relation to sensing with undetected light, and more carefully explain the study behind Fig. 4.

Fig.1 introduces an interferometer configuration that is familiar from other quantum and classical sensing with undetected photons or classical undetected light systems. Panels (f-i) show how interference at the camera breaks down when light is blocked at curved mirror 1, which represents sensing and detection both occurring at 580 nm, and

when light is blocked at curved mirror 2, which represents sensing at 1500 nm but detecting at 580 nm. The latter case is a binary sensing with undetected light measurement as it is a consequence of an interaction occurring exclusively with the seed (idler).

Fig. 2 looks at the visibility and phase of interference at 580 nm before and after placing a silicon window in the path of the seed (idler) beam (1500 nm) within the interferometer. We observe changes in visibility and phase, noting that pulse illumination and nonlinear reflections impose limitations that are key considerations for any interferometer using a thin-film nonlinear source, as discussed in detail in the main text. Importantly, silicon is completely opaque at 580 nm, meaning this result is necessarily a sensing with undetected light measurement, transferring information from the near infrared to the visible. Also, we would like to point out that the basic operation of an undetected photon/light scheme is to sense, and demonstration of such a scheme cannot be separated from the sensing it performs.

Fig. 3(e) and (f) together constitute the clearest demonstration of sensing with undetected light. The knife-edge experiment (e) shows the single spatial mode nature of the system which allows the knife edge itself to act as a variable transmission filter in the infrared path at the object plane. Our system senses changes in the transmission of the infrared path. The is retrieved from the interference contrast of the signal on a silicon detector. Moreover, we recover the expected square root dependence on transmission, which we compare with our theoretical model. Therefore, as requested by the reviewer, this experiment quantitatively evaluates the response of the system using a known reference sample.

Although the relatively broad bandwidth of the pulsed laser has clear applications to spectroscopy, the purpose of Fig. 4 was not to demonstrate a spectroscopic measurement of a sample. Instead, it experimentally confirms that the temporal alignment of the pump and seed outside the interferometer is decoupled from the spectral measurement performed within it. The aim was to show readers that the relative phases of the pump and seed pulses have nothing to do with the retrieved phase from the ilder part of the interferometer. The system measures the phase of an object independently of the delay of the two pulses. To show this, the delay line outside the interferometer was varied to show that the phase within the interferometer, Fig. 3(c), is invariant to the temporal overlap of the pulses. Furthermore, this figure highlights the potential to perform pulsed laser interferometry to be used for measuring intra-pulse dynamics of material placed in the idler arm, which was discussed in the first round of review. To help clarify the main point of this subsection it has been renamed:

“Invariance to temporal alignment”

Overall, we disagree that our manuscript does not demonstrate sensing using the developed system. In fact, Figs. 1-3 demonstrate sensing in the spatial domain to progressively greater degrees, culminating with Fig. 3 and the characterisation of the dependence of visibility on seed (idler) transmission using a variable transmission filter (knife edge) and theoretical verification of the response. This directly addresses the reviewer's request for *a* quantitative evaluation of the system's sensing response using a known reference sample. We acknowledge that Fig. 4 does not demonstrate spectral sensing of a sample, but this does not detract from its main purpose; to investigate the measurement's response to temporal alignment outside the interferometer, and the prospect of using this feature in future work.

Conversely, if the main goal is to emphasize characterization of the nonlinear interferometer itself rather than validation of sensing, then it would be important to experimentally verify the distinctive features of a metasurface-based nonlinear interferometer (aside from the side effect of bidirectional photon emission). At present, many of these features remain at the level of theoretical analysis and prediction. Taken together, the current proof-of-principle does not go beyond demonstrating the simple combination of a metasurface and a nonlinear interferometer.

A key goal of the work is characterisation of the distinct features of a nonlinear interferometer using a thin-film nonlinear source and pulsed illumination, which we have conducted in the context of a Michelson interferometer under normal-incidence excitation of the source. Our work experimentally verifies some critical considerations for building such a system: not just bi-directional nonlinear emission but also linear reflections from the source, temporal separation of interference, single-mode operation in the far field, non-interfering back-reflection, and invariance of the measurement (spectral or spatial) to temporal alignment. Regarding bi-directional emission specifically, we would argue this is not just a side effect but a defining experimental feature of thin-film nonlinear sources that distinguishes them from bulk crystal counterparts. Furthermore, the theoretical model of interference developed is in excellent agreement with the experimental measurements conducted. Building on these verified experimental foundations, we theoretically explore optimising visibility through metasurface engineering, isolating interfering fields through tilting the metasurface, and the alternative functionality of a Mach-Zehnder interferometer. As a first demonstration of nonlinear interferometry with a thin-film nonlinear source, this manuscript balances novel exhibition of the technique with promising avenues for future research. As nonlinear metasurfaces and thin films are of significant contemporary interest, this first demonstration represents a significant contribution to the field.

We respectfully contest the reduction of this work to a 'simple combination' of a thin-film metasurface and nonlinear interferometer, and disagree that their integration,

characterisation of the system, and validation of sensing can be treated as separate goals. Combining a thin-film source with nonlinear interferometry requires system characterisation that inherently involves sensing. Consequently, we have achieved all three of these goals. By integrating a thin-film source into a nonlinear interferometer and experimentally and theoretically characterising the system, we have demonstrated sensing with undetected light in the spatial domain, while drawing attention to non-trivial differences from conventional systems identified above.

【Other comments】

I recommend that the authors further clarify the four advantages they claim relative to the upconversion approach. At least in upconversion interferometry (e.g., optical coherence tomography), where the infrared light is sent through an interferometer and then upconverted, the (relative) phase of the sample can be measured and evaluated. If your argument is that interferometric measurement itself is what yields four advantages, then wouldn't an upconversion interferometer achieve essentially the same benefits as well?

The advantages the reviewer is referring to are in comparison to upconversion sensing, not involving an interferometer. We repeat this list of interferometric advantages here:

1. Phase sensitivity
2. Greater robustness to noise compared to a direct measurement of intensity
3. Improved sensitivity at low transmission in the idler arm due to the square root of transmission relationship
4. Immunity to thermal background noise and other incoherent background due to its coherent nature

What the reviewer has descripted as 'upconversion interferometry' is a version of nonlinear interferometry with the nonlinear source positioned outside the interferometer. However, the ability of the nonlinear interferometer to measure a samples phase is not dependent on the position of the source but, pragmatically, whether it is operated with a continuous-wave or pulsed laser. In essence, yes, an 'upconversion interferometer' would achieve the same benefits as it is effectively the same system.

Reponse to 3rd round review.

Comment from the editor: We have now received reports from 2 reviewers and, on the basis of their comments, we have decided to invite a revision of your work for further consideration in our journal. Your revision should address all the points raised by our reviewers (see their reports below). In particular, we would like you to tone down the claim of the wavelength range, as well as to discuss the potentials and limitations when extending the system to imaging.

Our Reply: Thank you for your consideration of our manuscript for publication in Nature Communications. We have addressed both concerns in our reply to the referees below.

Comment from the editor: When resubmitting, you must provide a point-by-point response to the reviewers' comments. Please show all changes in the manuscript text file with track changes or colour highlighting. If you are unable to address specific reviewer requests or find any points invalid, please explain why in the point-by-point response.

Our Reply: As you can see below, we have provided a point by point response to both of the remaining referees. Changes to the manuscript are shown under our replies in red and are also highlighted in the manuscript itself.

REVIEWER COMMENTS

Reviewer #1 (Remarks to the Author):

Reviewer #1 writes: The rebuttal letter by Millard et al. provides a convincing response to my first comment (about the visibility).

As to the response to my third comment (about the effect of the substrate), I am fully satisfied.

Our Reply: We would like to thank the referee for their continued patience and effort in the review of our work and for the significant improvement it has made to our manuscript.

Reviewer #1 writes: As to my second comment, about 'octave-spanning four-wave mixing', the authors still insist on the wording. The text now runs as "...we report octave-spanning classical sensing with undetected light using stimulated four-wave mixing from a plasmonic metasurface." They claim that 'as the sensing wavelength is 1500 nm and the detection wavelength is 580 nm, this spans almost two octaves and therefore can be described as octave spanning.'

I strongly disagree with this formulation. 'Spanning' means covering a range of frequencies. The fact that the detection wavelength and the sensing wavelength are much separated in spectrum is common for all 'undetected photons' techniques; however, none of the works speaks of 'octave-spanning sensing'. This is a misleading statement. The authors should choose a more careful way to indicate the large separation between the two wavelengths.

Our Reply: We appreciate the referee's concern on this matter. In the interest of absolute clarity, we have removed phrase 'octave-spanning', instead explicitly stating the range and the purpose of each wavelength. The first sentence in the abstract now reads:

In this article, we report classical sensing with undetected light at a wavelength of 1,500 nm, detected on a silicon camera at 580 nm, using four-wave mixing from a plasmonic metasurface.

Reviewer #3 (Remarks to the Author):

Reviewer #3 writes: I appreciate that the authors addressed as many of my concerns as possible. I already understand what is done in Figures 1-4, and from the response letter I also understand the authors' claim that observing changes in the amplitude or phase of light constitutes sensing in a broad sense; therefore, their verification is consistent with that claim. On the other hand, as I have mentioned in previous review comments, this paper does not actually acquire and evaluate images of objects with a two-dimensional distribution to demonstrate its applicability as an imaging technique, nor does it acquire and evaluate spectra of objects with different absorption/reflection for each frequency to demonstrate its applicability as a spectroscopic technique. Given that many other undetected-photons techniques acquire and evaluate images, spectra, phase images, or tomographic images of objects, I'm still not entirely convinced that this paper has reached the level required for publication in Nature Communications. This is because, within the scope of current verification, it is unclear which applications, including the aforementioned imaging and spectroscopy, this alone would be useful for. On the other hand, I understand their position that, because nonlinear interferometry using metasurfaces differs substantially from other methods, they should limit the verification here to what is minimally necessary for an undetected-photons technique, and that the aforementioned imaging or spectroscopy demonstrations should be the next step in a separate paper. I would like to leave the final decision on publication to the editor, taking these points into consideration.

Our Reply: We thank the referee for their patience throughout the review process as we have addressed their questions. Their comments have certainly improved the manuscript.

As stated in the title and throughout the manuscript, this work is about sensing – i.e. single mode response, and not about imaging. In terms of spectroscopy, we have reported spectrograms that encode the time delay between pulses and the spectral response, confirming that both phase and amplitude are unaffected by delay between pump and seed, and so also confirming that our system measures transmission phase and amplitude. We agree that we did not insert an object to sense spectrally, but the demonstration proves the function of the instrument incorporating a nonlinear metasurface.

We have added to the manuscript a comprehensive discussion on implementing imaging with our system, and those that might include any thin nonlinear material. This conveys the generality of the work and how it can be exploited in a variety of nonlinear materials. This will inform readers how imaging can be implemented with the system along with any advantages and limitations.

A seeded single-mode interferometer based on a phase matching free thin nonlinear medium can be extended to spatially resolved imaging via three routes. The most straightforward is to focus the seed (idler) to a diffraction-limited spot and raster-scan the object, providing high resolution at the cost of acquisition speed. Alternatively, by imaging the object plane directly onto the thin nonlinear medium and subsequently onto the camera, the near-field spatial distribution of the seed beam is encoded in the second-pass nonlinear emission and read out in the interference visibility. This bypasses the requirement for momentum correlations in the thin nonlinear medium and uses spatial correlations instead. While this enables parallel, and thus quicker, image acquisition, the image resolution and field of view would be limited by the aperture of the nonlinear medium. Multimode seeding offers the third route but inherits drawbacks of both approaches, offering both limited speed and resolution, while also demanding significantly higher power to seed the multiple modes. Finally, we note that with an efficient dielectric metasurface source, the interferometer could be operated via spontaneous nonlinear emission alone, with the biphoton state properties engineered directly through metasurface design.

Additionally, the summary towards to end of the manuscript has been edited to reflect the above change.

The single mode nature of interference, brought about by seeding the FWM with single mode lasers, provides no spatial information for imaging. However, the seed (idler) could be focused onto a scanned object plane to collect an image, additional modes

could be generated before the first pass through the nonlinear source, or spatial correlations of the FWM emission could be utilised.
